# How Can We Make Scientific Events More Inclusive? Insights From Q&A Sessions and Surveys From an International Conference

**DOI:** 10.1002/ece3.71588

**Published:** 2025-07-13

**Authors:** Rebecca S. Chen, Tuba Rizvi, Ane Liv Berthelsen, Anneke J. Paijmans, Avery L. Maune, Barbara A. Caspers, Bernice Sepers, Isabel Damas‐Moreira, Isabel Schnülle, Jana Könker, Joseph I. Hoffman, Joelyn de Lima, Jonas Tebbe, Kai‐Philipp Gladow, Lisa de Vries, Marc Gilles, Nadine Schubert, Nayden Chakarov, Peter Korsten, Petroula Botsidou, Sabine Kraus, Stephen M. Salazar, Svenja Stöhr, Wolfgang Jockusch, Öncü Maraci

**Affiliations:** ^1^ Department of Evolutionary Population Genetics Bielefeld University Bielefeld Germany; ^2^ Department of Evolutionary Biology Bielefeld University Bielefeld Germany; ^3^ Department of Behavioural Ecology Bielefeld University Bielefeld Germany; ^4^ Joint Institute for Individualisation in a Changing Environment (JICE) Bielefeld University and University of Münster Bielefeld Germany; ^5^ Department of Biochemistry and Physiology of Plants Bielefeld University Bielefeld Germany; ^6^ British Antarctic Survey Cambridge UK; ^7^ Center for Biotechnology (CeBiTec), Faculty of Biology Bielefeld University Bielefeld Germany; ^8^ The Teaching Support Center & Center for Learning Sciences The Swiss Federal Institute of Technology Lausanne Switzerland; ^9^ Department of Animal Behaviour Bielefeld University Bielefeld Germany; ^10^ German Institute for Adult Education Bonn Germany; ^11^ Department of Life Sciences Aberystwyth University Aberystwyth UK; ^12^ Berliner Akademie für Mediation Und Interkulturelle Kommunikation (BAMIK GmbH) Berlin Germany

**Keywords:** chilly climate, discrimination, diversity, inclusivity, question‐asking, underrepresentation

## Abstract

Despite growing awareness of the importance of researcher diversity, barriers to inclusion and equity persist in science and at academic conferences. As hosts of the 37th International Ethological Congress, “Behaviour 2023”, we studied gender disparities that unfold during question‐and‐answer (Q&A) sessions using observational and experimental behavioural data and surveys. We further used the surveys to investigate broader equity, diversity and inclusivity (EDI) issues at conferences in general. Attendees perceived as women asked fewer questions than those perceived as men because they raised their hands less often to ask questions, and not because they were chosen less often by the session host. Self‐reports indicated that self‐identified women felt more comfortable asking questions when their own gender was represented (in the audience, by the speaker, and/or by the host) and when the setting was smaller. However, this pattern was not reflected in the observational data as perceived women asked fewer questions regardless of the situation. We report potential reasons why women asked fewer questions using survey data, and experimentally tested whether we could reduce gender disparity in question‐asking. Our results indicate that session hosts cannot mitigate the gender disparity in question‐asking by actively selecting perceived women to start the Q&A session. We addressed further inclusivity barriers of underrepresented minorities beyond gender in a post‐congress survey, which showed that underrepresented minorities did not have a more positive or negative congress experience but did perceive EDI issues as more severe. We conclude by providing recommendations for organising more inclusive scientific events.

## Graphical abstract

We studied equity, diversity and inclusivity (EDI) issues at an academic conference using observational and experimental behavioural data collected during question‐and‐answer (Q&A) sessions in addition to surveys conducted before and after the congress. We identified multiple barriers that social minorities face during scientific conferences.

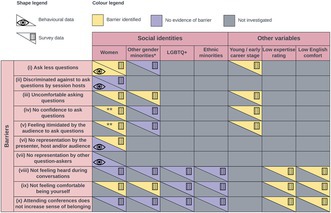

## 1 Introduction

Diversity within the scientific community is essential for advancing science because it facilitates the inclusion of a wide range of perspectives and contributions. There is growing evidence that increased gender and/or ethnic diversity can benefit science as a whole (Nielsen et al. 2017) by increasing productivity (Martin 2014; Saxena 2014), delivering higher quality science (Campbell et al. 2013), and producing papers with higher scientific impact (AlShebli et al. 2018). Despite these known advantages of researcher diversity in academia, the persistent lack of underrepresented minorities (groups of people whose representation in academia is lower compared to their representation in the general population) and ongoing inequities remain ubiquitous in academia, including in the biological sciences (Cronin et al. 2021; Lagisz et al. 2022; Lee 2020; McGill et al. 2021; Tulloch 2020).

### 1.1 Chilly Climates and Systemic Biases

Hostile or “chilly” academic climates are marked by discrimination (active or passive), harassment, microaggression and professional devaluation based on, e.g., sexism (Blithe and Elliott 2020; Casad et al. 2021; Clancy et al. 2014), racism (Martínez‐Blancas et al. 2023; McGee 2020; Settles et al. 2021), queerphobia (Cech and Waidzunas 2021; Marosi et al. 2024) and ableism (Brown and Leigh 2018; Cech 2023; Crabtree et al. 2023). Such environments can impede the entry, progress, and retention of marginalised groups in academia (Dorenkamp and Weiß 2018; Douglas et al. 2024; White‐Lewis et al. 2023), such as women, ethnic minorities, LGBTQ+ (lesbian, gay, bisexual, transgender, queer, and other identities under related to sexuality and/or gender) researchers, and individuals with disabilities, ultimately leading to a reduction in researcher diversity. The underrepresentation of senior women (Almukhambetova et al. 2023; Pell 1996; Resmini 2016) and senior scientists from other underrepresented minorities (Figueiredo 2024; O'Brien et al. 2020; Sarraju et al. 2023) has prompted initiatives to foster more inclusive academic climates, such as addressing stereotyping and discrimination, elevating the sense of belonging of minorities, ensuring safe workplaces, and institutional policy changes (McGill et al. 2021; Sardelis et al. 2017; Schell et al. 2020; Tilghman et al. 2021; Tseng et al. 2020).

### 1.2 Inclusivity Barriers Need to Be Identified to Provide Equitable Opportunities

Beyond individual discrimination, systemic biases in academic cultures and policies further disadvantage marginalised groups. Some groups might have different needs to ensure a healthy work‐life balance (e.g., due to caring duties; Hooker et al. 2017; Michailidis et al. 2012), gain access to mental health services (e.g., due to being more vulnerable to experiencing mental health issues, which is the case for LGBTQ+ scientists; Cech and Waidzunas 2021), ensure accessibility on campus or during fieldwork (e.g., due to disabilities; Miller and Downey 2020; Morales et al. 2020), or receive adequate mentorship (e.g., due to cultural differences; Womack et al. 2020). These aspects are essential to progress and excel, yet the focus is often put on distributing resources equally rather than equitably (Espinoza 2007; Secada 1989). Overcoming these biases requires proactive policy reforms rather than passive inclusion efforts. To effectively improve these policies and practices, we require a better understanding of existing barriers to inclusion and equity, for example by encouraging dialogue and observing what barriers unfold in natural settings.

### 1.3 Barriers to Inclusive Academic Conferences

Barriers to inclusion and equity also unfold at academic conferences. Conferences are crucial events for networking and gaining exposure, as they provide a space to connect with researchers with similar interests, promote one's own work (De Leon and McQuillin 2020) and collect information on jobs and funding opportunities, which are particularly important for early‐career researchers (Hauss 2021). However, certain groups of people can face barriers to invitation, participation or recognition at scientific conferences. For example, women and ethnic minorities are underrepresented as invited speakers (Bhayankaram and Prathivadi Bhayankaram 2022; Ford et al. 2018) and, on average, women receive a lower turnout at talks than men (Barreto et al. 2024; Lupon et al. 2021). High registration fees and travel expenses also create obstacles for researchers from low‐income countries, generating economic disparities. Additionally, factors such as a lack of proper accessibility to and within the conference venue (De Picker 2020), limited childcare options for caretakers, and the need for English proficiency can represent major barriers to ensuring an inclusive scientific conference.

### 1.4 Gender Disparities in Question‐Asking Probability

Research on equity, diversity and inclusivity (EDI) issues at conferences has grown, increasing awareness of common issues, yet knowledge gaps persist. A well‐documented issue is gender disparity in question‐asking, with studies showing that women ask fewer questions than men in Q&A (question‐and‐answer) sessions (Carter et al. 2018; Davenport et al. 2014; Hinsley et al. 2017; Käfer et al. 2018; Lupon et al. 2021; Pritchard et al. 2014), possibly due to a mix of factors like “not working up the nerve” or men asking the first question, which has consequences for the rest of the session (Carter et al. 2018). However, the causes and consequences of this disparity remain unknown, and there is, to our knowledge, no causal evidence of what actions could encourage women to ask more questions. In addition to gender disparities in question‐asking behaviour, the exact barriers that certain social identities face at conferences must be identified. Barriers can arise from discrimination, prejudice and/or a tendency to dismiss specific contributions, which all play a role in forming a “chilly conference climate”, negatively impacting the experience of those affected. It is therefore crucial to improve our current understanding of contemporary EDI‐related issues that occur at scientific conferences to be able to organise more inclusive events.

### 1.5 Inclusivity at Behaviour 2023

To address the knowledge gaps highlighted above, we conducted a comprehensive study during the 37th International Ethological Congress, ‘Behaviour 2023’, hosted at Bielefeld University, Germany. The congress focused on animal behaviour and was attended by delegates from a range of backgrounds, including ethology, ecology, evolutionary biology, behavioural genetics and anthropology. This congress therefore provides an excellent opportunity to understand EDI issues, as it was attended by a diverse group of people with regard to their educational background, (work) culture, and career stage, therefore not biasing observations towards a specific institute or group. We used a combination of observational and experimental data collected from three different sources: (i) congress registration (quantitative self‐reports regarding attendees' social identities; 727 responses), (ii) Q&A sessions (quantitative observational data regarding perceived gender disparities in question‐asking probability; 1278 questions asked in 67 sessions), and (iii) a post‐congress survey (quantitative self‐reports regarding congress experiences and perceptions of EDI issues as well as qualitative feedback; 391 responses). In 40 out of 67 Q&A sessions, we experimentally tested whether session hosts can increase the probability that women ask questions by instructing them to either direct the first question after a talk to a male or female participant. Combining qualitative and quantitative data allowed us to gain a deeper understanding of key inclusivity issues related not only to gender identity but also to nationality, sexual orientation, and disability.

## 2 Results

We investigated various aspects of inclusivity and inequality among social identities at a scientific event using a case study. The congress took place in August 2023 and was attended by 855 people. The language of the congress was English, and a total of 661 oral presentations were given, distributed across continuous parallel sessions, as well as 11 plenary talks presented by speakers invited by the organising committee. The organising committee of the congress took a number of measures to boost inclusivity at the congress, including recommendations made by Joo et al. (2022), such as consciously counteracting biases in the conference programme and providing safe spaces (see Section 4 for details).

First, we report the social identities of congress attendees (Section 2.1). Second, we test for a gender disparity in question‐asking behaviour and address if there are practical measures that can be taken to mitigate the gender disparity (Section 2.2, 2.3, 2.4, 2.5). Third, we identify barriers that affect the conference experience of researchers with different social identities (Section 2.7, 2.8, 2.9).

### 2.1 What Social Identities Were Present at the Congress?

Prior to testing for barriers to inclusive conferences, we assessed the diversity of conference attendees. A total of 727 attendees took part in the pre‐congress survey, a subsection of the online congress registration form, which gathered data on the social identities of congress attendees. 65% of the attendees who provided their pronouns used she/her, and 33% used he/him. Fifteen attendees (2%) used she/they, he/they or they/them pronouns. 14.4% of attendees who responded to the question ‘if they identified with the LGBTQ+ community’ responded with “yes” (*n* = 92). Fifty‐nine nationalities were represented among the congress attendees. The majority of attendees who filled in details on their nationality were of European nationality (*n* = 481), followed by Asian (*n* = 85), North American (*n* = 48), Oceanic (*n* = 20), South American (*n* = 18), and African nationalities (*n* = 5). Most of the attendees with European, North American and Oceanic nationalities used she/her pronouns, but the majority of Asian and South American attendees used he/him pronouns. Lastly, four people acknowledged the need for some form of assistance during the congress due to either physical or mental disabilities.

### 2.2 Is There a Gender Disparity in Question‐Asking Probability?

Next, we tested whether women asked fewer questions than men using two lines of evidence based on (i) observational data on question‐asking behaviour collected during Q&A sessions after oral presentations (388 questions asked after 134 unmanipulated talks that were not part of our experiment, see below) and (ii) self‐reports on question‐asking collected in the post‐congress survey (373 complete responses).

#### 2.2.1 Gender Disparity During Q&A Sessions

To identify a gender disparity in question‐asking probability using the observational data, we investigated whether fewer questions are asked by perceived women. We categorised congress attendees as woman, man or ‘other’ (e.g., non‐binary) based on their appearance (following e.g., Carter et al. 2018; Hinsley et al. 2017) and/or their pronouns printed on their name tag (if available and readable). We recognise that perceived gender does not always correspond to self‐identified gender, which is especially true for non‐binary individuals (see Section 4.3 for details). However, discrimination ultimately acts on the perception of a person's identity, regardless of whether the identity has been disclosed (Quinn and Earnshaw 2013). To better understand the experience of non‐binary individuals during Q&A sessions, we use the post‐congress survey where respondents self‐identified their gender (see the Section 2.2.3 below). In all the sections that use the observational data collected during Q&A sessions, we abbreviate “perceived gender” to “gender” for readability but clarify how gender was quantified (perceived versus self‐identified) when specifying the statistical models in each paragraph.

**FIGURE 1:**
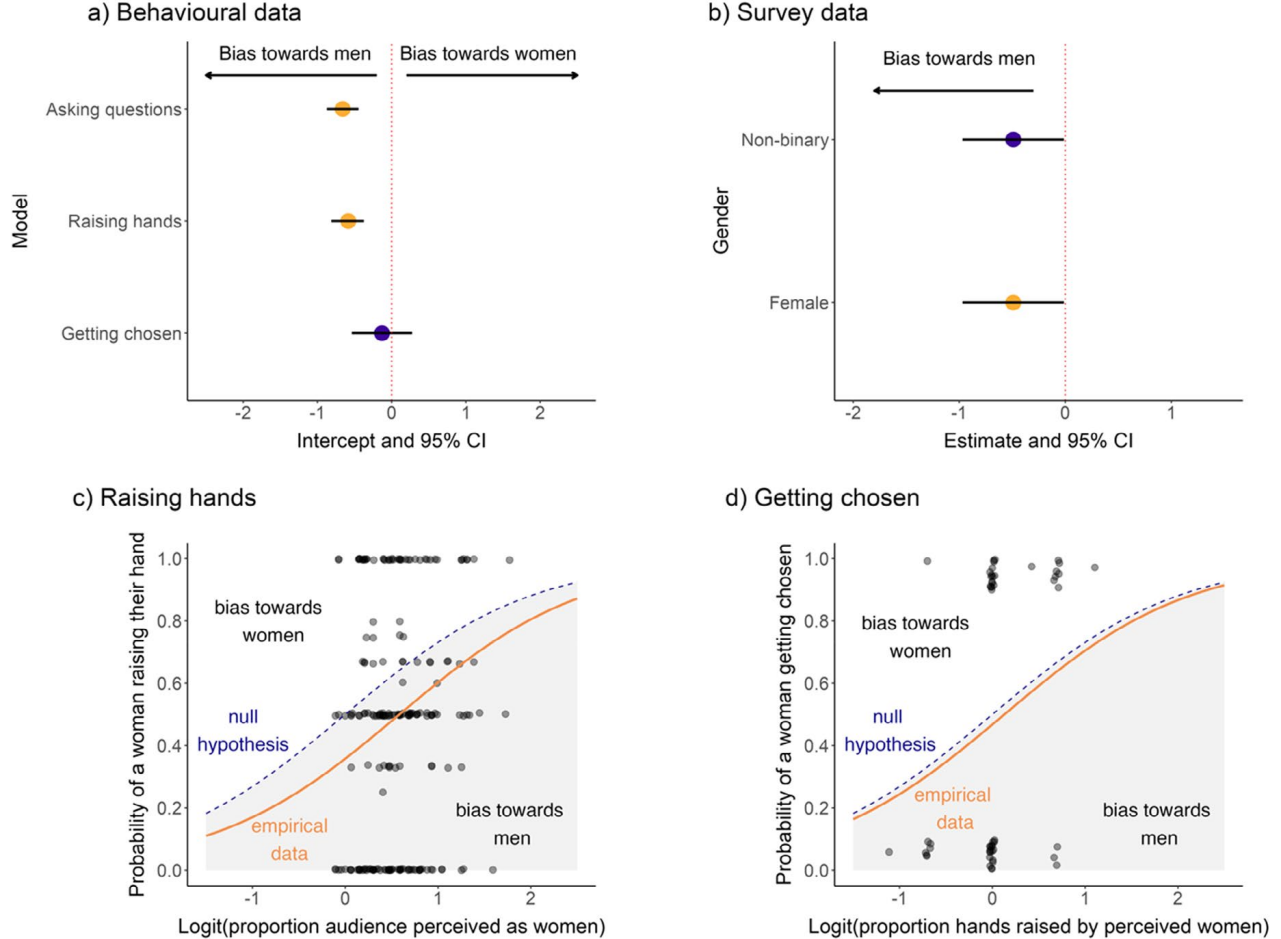
Gender disparity in question‐asking behaviour. (a) Intercepts and 95% confidence intervals (CI) for models QA.1, QA.2 and QA.3 that tested for perceived gender disparities in asking questions, raising hands and being chosen, respectively. Yellow points indicate statistically significant intercepts (*p* < 0.05). A negative intercept indicates that the probability that a perceived woman asked a question was lower than expected from the number of women in the audience (bias towards men), whereas a positive intercept indicates that this probability was higher than expected (bias towards women). (b) Model estimates and 95% CIs for the effect of self‐identified gender on the probability of asking a question based on the survey data. Yellow points indicate statistically significant effects (*p* < 0.05). A negative estimate indicates that the gender in question is negatively associated with the probability that a woman asked a question (i.e., positively associated with the probability that a man asked a question; a male bias). (c) Raw data with added jitter, null hypothesis and model estimates for model QA.2, which tested for a disparity in perceived gender for raising hands; (d) Raw data with added jitter, null hypothesis (no gender disparity) and model estimates for model QA.3, which tested for a disparity in perceived gender for being chosen to ask a question.

To assess whether women asked fewer questions than men, we fitted a binomial generalised linear mixed effect model (GLMM), where the dependent variable indicates whether a question was asked by a perceived man (0) or a perceived woman (1). In the model, we accounted for the perceived gender proportion of the audience and the non‐independence of talks within a session (see Section 4 for details). Across all unmanipulated talks, 48% of questions were asked by women without accounting for the proportion of women in the audience. The overall probability that a woman asked a question, corrected for the proportion of women in the audience, was 0.34 (GLMM intercept = −0.66, *p* < 0.001, Figure 1a; Table S1). An intercept significantly different from zero (*p* < 0.05) here indicates that the probability that a woman asked a question was different from 0.50. This result therefore provides clear evidence that women are less likely to ask questions compared to men.

When repeating the analysis with a more conservative dataset that excluded questions where the observer noted any source of uncertainty in the data collected, the results remained virtually identical (GLMM intercept = −0.67, *p* < 0.001; Table S1). Moreover, the perception of a congress attendee's gender might be biased by the observer's gender or ethnic background. When repeating the analysis with a dataset that only includes sessions observed by multiple people and data where all observers agreed on the attendee's gender, the conclusions remained the same (GLMM intercept = −0.43; *p* = 0.02; Table S2). Excluding sessions observed by only one person drastically reduced the dataset and therefore statistical power. We therefore include all observed sessions, regardless of how many observers were present, for the rest of the analyses.

#### 2.2.2 Gender Disparity During Plenary Q&A Sessions

We further collected observational data on question‐asking behaviour during plenary talks. We analysed these data separately from the other oral presentations because these sessions were held in larger lecture rooms attended by the vast majority of congress participants. We did not correct for the proportion of perceived women in the audience because it was unfeasible to count the audience by eye due to lack of visibility, size of the room, and difficulty keeping track of which people were and which people were not already counted. Consequently, we corrected for an estimated proportion of women in the audience by using the proportion of congress registrants that used she/her pronouns rather than correcting for audience counts, while correcting for plenary ID using a random effect. A total of 60 questions were asked during 11 plenary talk Q&A sessions, 17 (28%) of which were asked by women. Despite this relatively small sample size, the gender disparity in question‐asking was even greater during plenary sessions compared to regular oral presentations, with the probability of a woman asking a question being only 0.20 when correcting for the estimated proportion of people using she/her pronouns in the audience (GLMM intercept = −1.54, *p* < 0.001; Table S1).

#### 2.2.3 Gender Disparity in Question‐Asking Using Self‐Reports

Similarly, we tested for a gender disparity in question‐asking probability using self‐reports from the post‐congress survey, where we asked if fewer self‐identified women asked questions. We fitted a binomial generalised linear model (GLM) using the binomial response to the question “Did you ask a question at the congress?” (1 = yes, 0 = no) as the dependent variable and the self‐reported gender identity (woman, man, non‐binary, other) as the independent variable. Note that this question therefore addresses the likelihood that a woman asked a question across the entire congress, as opposed to the observational data that addresses if questions were less likely to be asked by perceived women based on each talk. Although including gender in the model barely improved the model fit (likelihood‐ratio test (LRT) *p* = 0.05), the results again showed that women were less likely to have asked a question during the congress compared to men, with the probability of a woman having asked a question being 0.38 (*beta* estimate female = −0.49, *p* = 0.04; Figure 1b; Table S1), while non‐binary people (*n* = 7) were not more or less likely to ask a question compared to men (probability = 0.71, *beta* estimate non‐binary = 0.92, *p* = 0.40; Figure 1b; Table S1).

### 2.3 Do Women Raise Their Hands Less Often or Get Chosen to Ask a Question Less Often?

#### 2.3.1 Do Women Raise Their Hands Less Often?

We next tested whether women asked fewer questions than men did because they raised their hands less often to ask a question using the observational data. We fitted a multivariate binomial GLMM where the dependent variable was the fraction of perceived women who raised their hands over the total number of people who raised their hands, while accounting for the proportion of perceived women in the audience and the non‐independence of talks within a session (see Section 4 for details). The probability that a woman raised their hand was 0.36 (intercept = −0.58, *p* < 0.001; Figure 1a,c; Table S1), indicating that women were less likely to raise their hand than men were.

#### 2.3.2 Do Women Get Chosen to Ask a Question Less Often?

Additionally, women might be chosen less often to ask their questions by the session hosts when both women and men raise their hands. We tested this hypothesis by fitting another binomial GLMM using the perceived gender of the questioner as the dependent variable, but this time correcting the proportion of the people who raised their hands that were perceived as women. In this model, we only included cases where at least one perceived woman and one perceived man raised their hand so that the session host had to make a choice between assigning the question to one gender over the other. The probability that a woman was chosen to ask their question by the session host was 0.46, indicating that women were not chosen significantly less often by session hosts to ask their question compared to men (intercept = −0.14, *p* = 0.53; Figure 1a,d; Table S1). We investigated this same question using the post‐congress survey data, where we collected data on a person's gender and whether one of the reasons they did not ask a question was due to not being chosen despite raising their hand (“not being chosen” in short). We fitted a binomial GLM with the response to “not being chosen” as the dependent variable and self‐reported gender as the independent variable. Including gender in the model did not significantly improve the model fit (LRT *χ* ^2^ = 1.49, LRT *p* = 0.47), indicating that women were equally likely to be chosen to ask their question, in line with our results based on the observational data.

### 2.4 Why Do Women Ask Fewer Questions Than Men Do?

#### 2.4.1 Are Gender Minorities Less Comfortable Asking Questions?

Next, we tested whether self‐identified women and self‐identified non‐binary respondents were less comfortable asking a question using data collected in the post‐congress survey. Respondents of the survey indicated their agreement to the statement “I feel comfortable asking questions during Q&A sessions” on a 7‐point Likert scale (1 = “Strongly disagree”, 7 = “Strongly agree”). We fitted an ordinal logistic regression model (OLR) for the response to this statement and included the self‐reported gender as an independent variable while correcting for career stage (early, mid or late career). We found that both women (*beta* estimate = −1.26, SE = 0.21, *t* = −5.99, *p* < 0.001) and non‐binary respondents (*beta* estimate = −1.60, SE = 0.68, *t* = −2.36, *p* < 0.02) felt less comfortable asking questions during Q&A sessions compared to men. Both mid‐career (*beta* estimate = 1.04, SE = 0.21, *t* = 5.04, *p* < 0.001) and late‐career researchers (*beta* estimate = 2.48, SE = 0.33, *t* = 7.50, *p* < 0.001) were more comfortable asking questions compared to early‐career researchers.

#### 2.4.2 Are There Gender‐Biased Motivations and Hesitations to Ask Questions?

The post‐congress survey additionally included questions on what aspect(s) motivated people to ask questions at the Behaviour 2023 conference (hereafter referred to as “motivations”) and what aspect(s) made people more hesitant to ask a question (hereafter referred to as “hesitations”). We tested in two steps if self‐identified women asked fewer questions than self‐identified men because they had different motivations and hesitations to ask questions than men did. The first step tested which motivations and hesitations were more often selected by self‐identified women compared to self‐identified men. We fitted multiple binomial GLMs, one per motivation and hesitation. In each case, the dependent variable was the binomial response whether the motivation or hesitation was ticked (1) or not (0), and the independent variables were self‐reported gender and career stage (early, mid or late career). The second step tested which of the motivations and hesitations that were significantly affected by gender were significant predictors of the probability of a person asking a question during the congress, where we then examined which of the significant ones were also affected by self‐identified gender. We fitted a second set of binomial GLMs, again one for each motivation and hesitation. The dependent variable in these models was the response to the question, “Did you ask one or more questions during Q&A sessions?” (1 = yes, 0 = no), and the independent variable was the binomial response whether the motivation or hesitation was ticked (1) or not (0), while also including gender and career stage as covariates.

**FIGURE 2:**
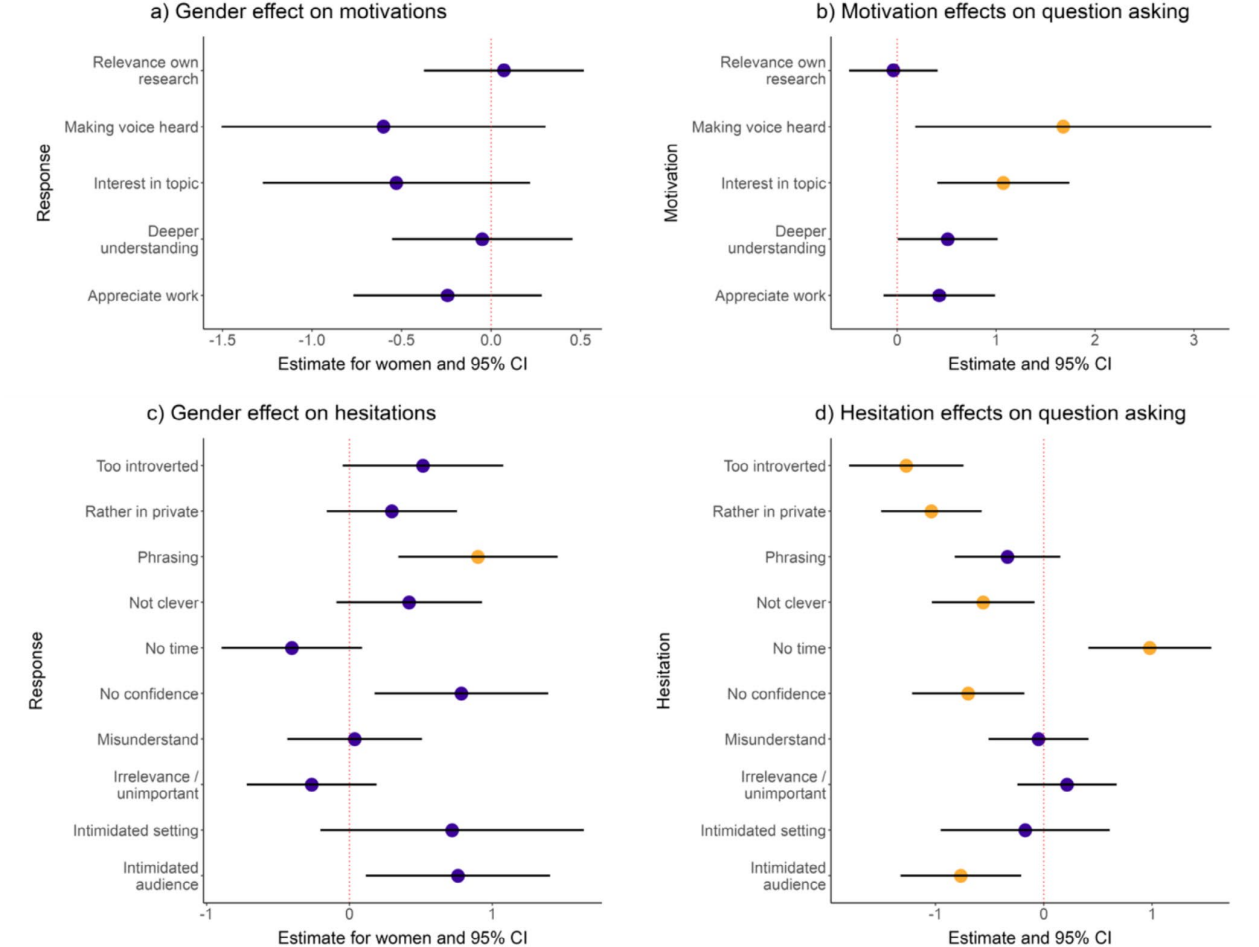
The results of models that tested for gender disparities in question‐asking due to different motivations and hesitations between women and men. Model estimates of the effect of self‐identifying as a woman on (a) six motivations and (b) the effects of the motivation on the probability that the person asked a question during the congress, as well as the effect of self‐identifying as a woman on (c) 12 hesitations and (d) the effects of the hesitation on the probability that the person asked a question during the congress. Yellow points indicate including the variable in the model significantly improved the model fit compared to the null model after correcting for multiple testing.

Including self‐identified gender as an independent variable did not improve the fit of any of the models fitted to the motivations (FDR‐corrected LRT *q* < 0.05; Figure 2a, Table S2), indicating that women were not more likely to select any of the motivations compared to men. However, when looking at the hesitations, “afraid I would not be able to phrase/articulate my question well” was significantly affected by gender after correcting for multiple testing, where women were more likely to tick this hesitation compared to men (*beta* estimate for women = 0.90, *p* for women = 0.002, Figure 2c, Table S3). Two more hesitations were affected by gender but were not statistically significant after correcting for multiple testing: “I did not have the confidence” (*beta* estimate for women = 0.78, *p* = 0.01, FDR‐corrected LRT *q* = 0.08; Figure 2c, Table S3) and “I felt intimidated by the audience” (*beta* estimate for women = 0.76, *p* = 0.02, FDR‐corrected LRT *q* = 0.13; Figure 2c, Table S3). For all of the other hesitations, the inclusion of gender did not improve the fit of the models (LRT FDR‐corrected *q*‐value < 0.05; Figure 2c, Table S3). Early‐career researchers were more likely to tick almost all hesitations compared to mid‐ and late‐career researchers (Table S4).

We found that most of the motivations and hesitations that were predictive of question‐asking probability were not influenced by self‐identified gender (Figure 2b, Figure 2d, Tables S2 and S3). The only hesitation that varied significantly by gender (fear of the inability to phrase/articulate a question well) did not influence the probability of asking a question during the congress (*beta* estimate = −0.34, *p* = 0.18; Figure 2d, Table S3). However, the two hesitations that were associated with gender only before applying a multiple‐testing correction (lack of confidence and feeling intimidated by the audience) were significant predictors of the probability of asking a question (lack of confidence: *beta* estimate = −0.70, *p* = 0.008, FDR‐corrected LRT *q* = 0.02; feeling intimidated by the audience *beta* estimate = −0.77, *p* = 0.07, FDR‐corrected LRT *q* = 0.02; Figure 2d, Table S3). Taken together, these results suggest that women are more likely to indicate that they are hesitant to ask a question because of a lack of confidence and/or feeling intimidated by the audience compared to men, which may make them less likely to ask a question, although not significant after multiple‐testing correction.

### 2.5 What Conditions Might Encourage Women to Ask Questions?

#### 2.5.1 Do Women Ask More Questions Under Certain Conditions?

**FIGURE 3:**
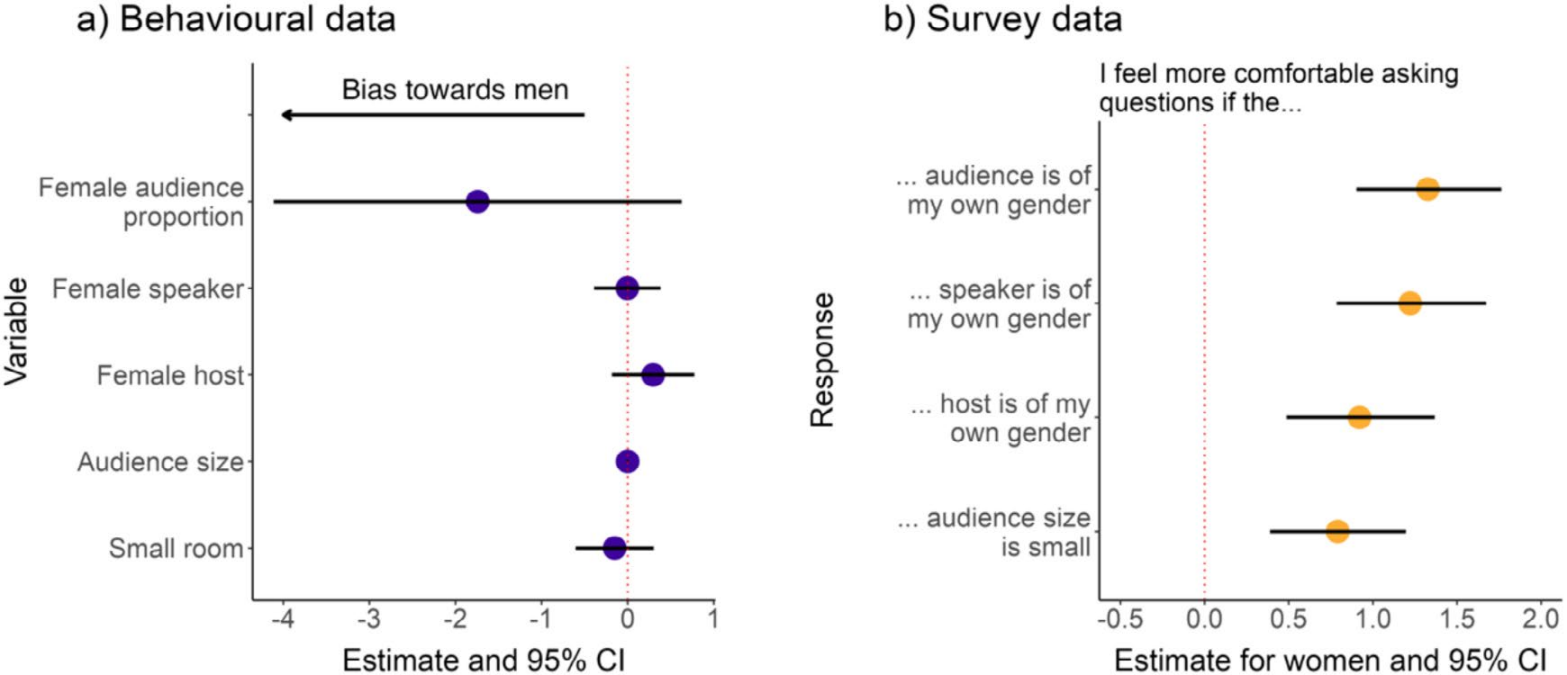
The results of models that evaluated what conditions can encourage women to ask questions. (a) Model estimates and 95% CI for the effect of five variables on the probability of a perceived woman asking a question based on the behavioural data. A negative estimate indicates that the variable in question was negatively associated with the probability that a perceived woman asked a question (i.e., it was positively associated with the probability that a man asked a question; a bias towards men). Yellow points indicate that including the variable in the model significantly improved the model fit compared to the null model. (b) Model estimates and 95% CIs for the effect of self‐identifying as a woman on the Likert‐scale response of four statements asked in the post‐congress survey. Yellow points indicate a statistically significant effect of female gender (*p* < 0.05).

We investigated which conditions might reduce the gender disparity in question‐asking probability. First, we tested which of the following five variables significantly affected the probability of a perceived woman asking a question based on the observational data: (i) speaker's perceived gender, (ii) perceived gender proportion of the audience, (iii) host's perceived gender, (iv) total audience size, and (v) room size. We fitted five binomial GLMMs for the probability that a perceived woman asked a question with one of the five variables as an independent variable, while correcting for the perceived gender of the audience and the non‐independence of talks within a session. None of the five factors significantly improved the fit of the models, indicating that they did not significantly affect the probability that a woman asked a question (LRT *p* > 0.05 for all five GLMMs, Figure 3a; Table S5).

#### 2.5.2 Are Women More Comfortable Asking Questions Under Certain Conditions?

Next, we addressed the same question using data collected in the post‐congress survey, addressing whether self‐identified women but also self‐identified non‐binary participants (despite low sample size, *n* = 7) were more or less comfortable asking questions in particular situations compared to self‐identified men. We asked respondents to indicate on a 7‐point Likert scale to what extent they agree with the following five statements: “I feel more comfortable asking a question if…” (i) “… the presenter is of my own gender”, (ii) “… there is representation of my gender in the audience”, (iii) “… the host is of my own gender”, (iv) “… the audience size is smaller”, and (v) “… if I know the speaker”, partially reflecting the variables described above. We fitted four OLR models, with the Likert‐scale response to each of the five questions as the dependent variable and self‐reported gender identity and career stage as independent variables.

Including self‐identified gender in the model improved the fit of almost all models (Figure 3b; Table S6), where women and non‐binary participants felt more comfortable asking questions compared to men when the speaker was of their own gender (women: *beta* estimate = 1.23, *p* < 0.001; non‐binary: *beta* estimate = 2.44, *p* < 0.001), their own gender was represented in the audience (women: *beta* estimate = 1.34, *p* < 0.001; non‐binary: *beta* estimate = 1.90, *p* < 0.01), or the host was of their own gender (women: *beta* estimate = 0.93, *p* < 0.001; non‐binary: *beta* estimate = 1.58, *p* = 0.02). Only women felt more comfortable than men asking questions when the audience size was smaller (women: *beta* estimate = 0.79, *p* < 0.001; non‐binary: *beta* estimate = −0.07, *p* = 0.91). Compared to men, neither women nor non‐binary people felt more or less comfortable asking questions when they knew the speaker (women: *beta* estimate = 0.92, *p* = 0.12; non‐binary: *beta* estimate = −0.19, *p* = 0.78).

### 2.6 Can Session Hosts Mitigate the Gender Disparity in Question‐Asking?

#### 2.6.1 Do Women Ask More Questions if Another Woman Started the Q&A?

Previous correlational research has shown that women can be encouraged to ask questions if a woman asks the first question in a Q&A session (Carter et al. 2018). We used observational data to test for this pattern in our data by quantifying the effect of the perceived gender of the first questioner on gender disparities in question‐asking in the rest of that session. More specifically, we fitted three binomial GLMMs to test for an effect of the perceived gender of the person who started the Q&A on the probability that (i) a question was asked by a perceived woman, corrected for the proportion of perceived women in the audience; (ii) a perceived woman raised her hand, corrected for the proportion of perceived women in the audience; and (iii) a perceived woman was chosen by the session host to ask her question, corrected for the proportion of people who raised their hand who were perceived women. The models had a nearly identical structure to the three models presented in Methods Sections ii and iii but included an additional fixed effect of the gender of the first questioner, and we removed the intercept for easier interpretation of the model output.

**FIGURE 4:**
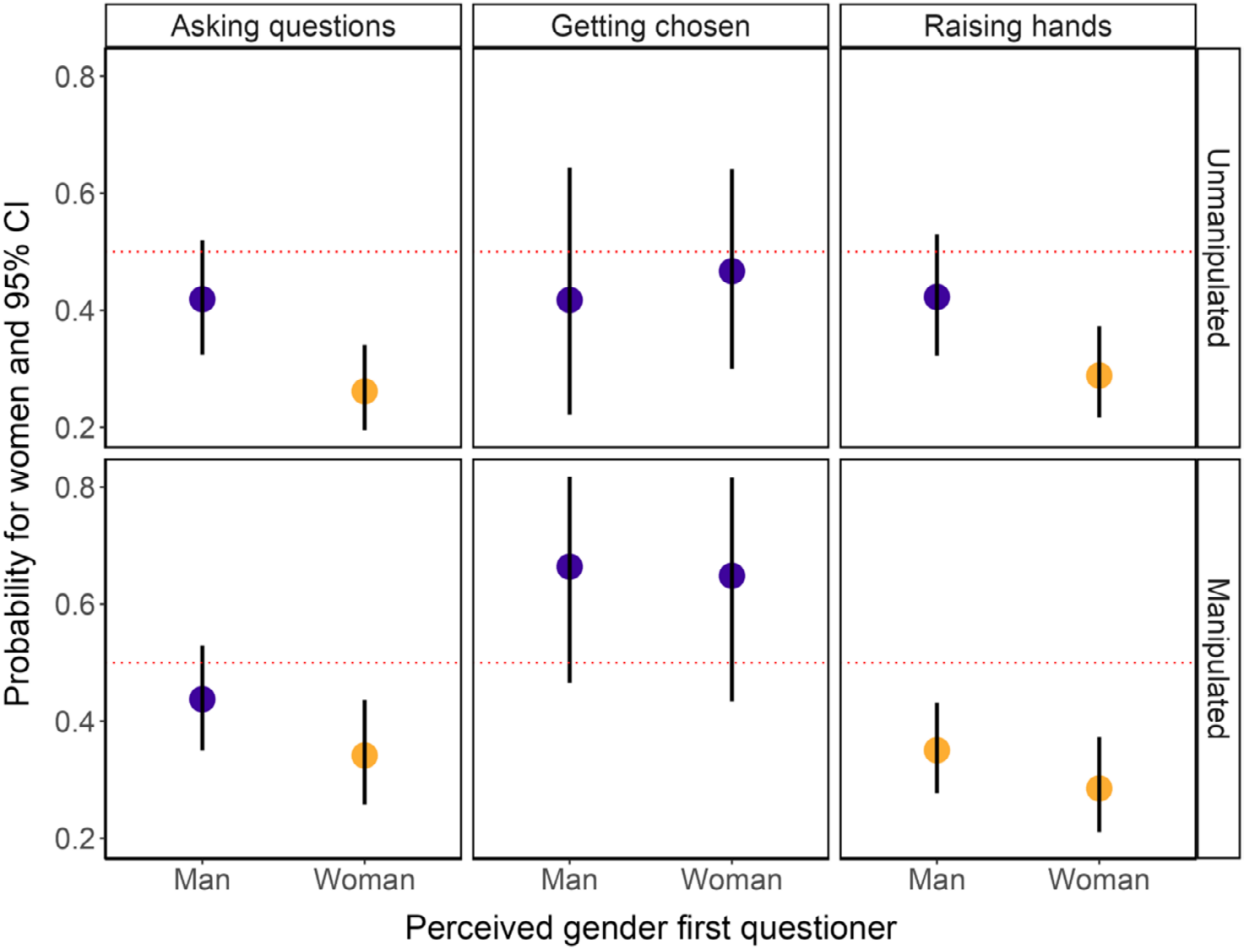
Model results showing the effect of the perceived gender of the first questioner on question‐asking probability. Points indicate the probability that a perceived woman asked a question, raised their hand, and was chosen to ask a question (left to right) for the unmanipulated and manipulated sessions. Yellow points indicate statistical significance (*p* < 0.05).

The perceived gender of the first questioner significantly affected the probability of perceived women asking a question (LRT *p* = 0.01, Table S7). Indeed, women were less likely than men to ask a question after a woman started the Q&A (*beta* estimate = −1.04, *p* < 0.001; Figure 4; Table S7), but not after a man started the Q&A (*beta* estimate = −0.33, *p* = 0.12; Figure 4; Table S7). Similarly, the gender of the first questioner significantly affected the probability of women raising their hands (LRT *p* = 0.03), as women were less likely to raise their hand than men after a woman started the Q&A (*beta* estimate = −0.90, *p* < 0.001; Figure 4; Table S7), but not when a man started the Q&A (*beta* estimate = −0.31, *p* = 0.16; Figure 4; Table S7). The gender of the first questioner did not significantly affect the probability of a woman being chosen to ask a question (LRT *p* = 0.74), as women were not significantly more or less likely to get chosen than men, regardless of whether a woman (*beta* estimate = −0.13, *p* = 0.72; Table S7) or a man (*beta* estimate = −0.33, *p* = 0.48; Figure 4; Table S7) started the Q&A. Similar results were obtained for all three models when testing for the effect of the gender of the first questioner on the probability of a woman asking the second question only (Table S8).

#### 2.6.2 Can Experimental Manipulation of Host Choice Close the Gender Disparity in Question‐Asking?

We sought to find causal insights into the effect of the gender of the first questioner by conducting an experiment in which we manipulated host behaviour. In the experiment, session hosts were instructed to either give the first question in the Q&A session to a woman or to a man. This manipulation allowed us to directly evaluate whether the perceived gender of the first questioner affected the probability of perceived women asking questions subsequently, regardless of the dynamics between the audience's behaviour and the session host's choice. The same models as described above were fitted using data collected from the successfully manipulated talks.

The perceived gender of the first questioner did not significantly affect the probability of a perceived woman asking a question, raising their hand or being chosen to ask a question in the sessions where the host choice was manipulated (LRT all *p* > 0.13, Table S7). Indeed, women were always less likely to ask a question than men, although this difference was only significant after a woman started the Q&A (*beta* estimate = −0.66, *p* = 0.001; Figure 4; Table S7) but not after a man started the Q&A (*beta* estimate = −0.25, *p* = 0.18; Figure 4; Table S7). Women always raised their hands significantly less often than men, regardless of whether a woman (*beta* estimate = −0.92, *p* < 0.001; Figure 4; Table S7) or a man started the Q&A (*beta* estimate = −0.62, *p* < 0.001; Figure 4; Table S7). Finally, women were not chosen to ask their question more or less often than men were, regardless of whether a woman (*beta* estimate = 0.61, *p* = 0.17; Figure 4; Table S7) or a man started the Q&A (*beta* estimate = 0.68, *p* = 0.10; Figure 4; Table S7). Interestingly, if we only selected the second question in each session, we found that women were significantly less likely to raise their hand than men after a woman started the Q&A (*beta* estimate = −0.93, *p* = 0.003; Table S8) but not after a man started the Q&A (*beta* estimate = −0.32, *p* = 0.28; Table S8).

### 2.7 How Did People With Different Social Identities Experience the Congress?

In the post‐congress survey, we asked respondents to indicate their agreement with the following three statements on a 7‐point Likert scale:

1. “I felt heard during the conversations I had, both during Q&A sessions and social activities” (“feeling heard” in short)
2. “I felt comfortable being myself” (“comfortable being myself” in short)
3. “Attending the Behaviour 2023 congress helped me feel like I belong in my research field” (“sense of belonging” in short)

We tested which of the following social identity variables were associated with the response to each of the three statements: self‐identified gender, LGBTQ+, nationality (continent), affiliation (continent), and expatriate status (“expat” in short, defined as a person whose country of affiliation was different from the country of their nationality). Expatriate status was included because research has shown that expatriation for work helps the development of cultural intelligence (Morin and Talbot 2023), which is “the capability for success in new cultural settings” (Earley and Ang 2003), which we would expect to play an important role at international scientific events. Additionally, we tested for the effects of the level of comfort a person had speaking English (“English comfort”), which reflects a combination of factors, including social environments, culture, and socio‐economic status that affect one's English language proficiency, as well as fear and anxiety to use the language (Janardhan 2024; Khan et al. 2024). We further tested for a person's self‐reported level of expertise (“expertise rating”), which is highly correlated with age (*beta* estimate for ages 35–50 = 2.02, *p* < 0.001; *beta* estimate for ages > 50 = 3.43, *p* < 0.001) and career stage (*beta* estimate for mid‐career stage = 2.21, *p* < 0.001; *beta* estimate for late‐career stage = 4.16, *p* < 0.001) but also captures variation in confidence.

**FIGURE 5:**
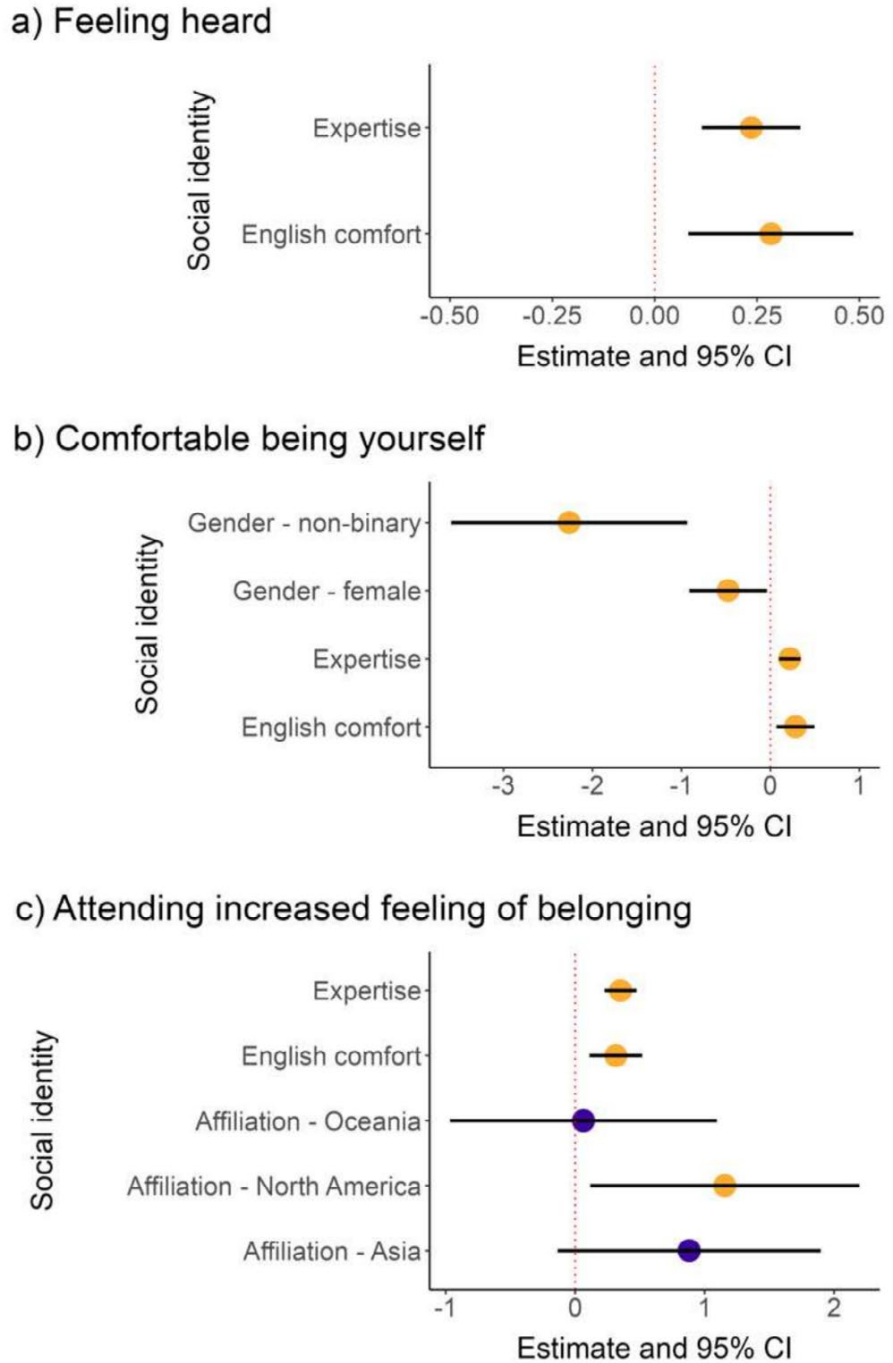
The results of models evaluating which social identities were significantly associated with variation in congress experiences. (a) Model estimates and 95% CIs of the final model that tested for the effect of social identity variables on the Likert‐scale response to the statement on feeling heard at the congress. (b) Model estimates and 95% CIs of the final model that tested for the effect of social identity variables on the Likert‐scale response to the statement on feeling comfortable being yourself. (c) Model estimates and 95% CIs of the final model that tested for the effect of social identity variables on the Likert‐scale response to the statement on congress attendance increasing one's feeling of belonging in the research field. The reference continent for affiliation to which the other continents were compared was Europe. The estimates and 95% CIs for African and South American affiliations on statement (c) (Table S9) were excluded due to small sample sizes. Yellow points indicate a statistically significant effect of the social identity variable in the final models.

First, we fitted one univariate OLR model per statement and per social identity. If including the social identity in the univariate model significantly improved model fit, assessed with an LRT, we included the variable in the final model for that statement. We found that people with higher agreement to the “feeling heard” statement also felt more comfortable speaking English (*beta* estimate = 0.28, *p* = 0.006; Figure 5a; Table S9) and rated themselves as having a higher level of expertise in their field (*beta* estimate = 0.24, *p* < 0.001; Figure 5a; Table S9). Similarly, people with higher agreement to the “comfortable being myself” statement also felt more comfortable speaking English (*beta* estimate = 0.28, *p* = 0.01; Figure 5b; Table S9) and rated themselves as having a higher level of expertise in their field (*beta* estimate = 0.22, *p* < 0.001; Figure 5b; Table S9). Moreover, women and non‐binary people felt less comfortable being themselves (*beta* estimate women = −0.48, *p* women = 0.03; *beta* estimate non‐binary = −2.26, *p* non‐binary = 0.001; Figure 5b; Table S9) compared to men. Lastly, people with higher agreement to the “sense of belonging” statement also felt more comfortable speaking English (*beta* estimate = 0.31, *p* = 0.002; Figure 5c; Table S9) and rated themselves as having a higher level of expertise in their field (*beta* estimate = 0.36, *p* < 0.001; Figure 5c; Table S9). People with a North American affiliation had higher agreement to “sense of belonging” compared to those with a European affiliation (*beta* estimate = 1.16, *p* = 0.03); however, we interpret any effects of affiliation with care due to variation in sample sizes, as only 19 North American affiliates filled in the post‐congress survey as opposed to 334 European affiliates.

#### 2.7.1 Did Conference Attendees Experience Discrimination and/or Harassment?

Respondents to the post‐congress survey were also asked if they experienced discrimination and/or harassment (of any sort) at the congress and whether they reported it to the awareness team or if they witnessed someone else experiencing this. A total of 11 respondents reported experiencing some form of discrimination or harassment, of which two cases were reported to the awareness team. Eight of the 11 cases were reported by women, two by men, and six by LGBTQ+ and/or non‐binary attendees. A total of three survey respondents witnessed somebody else experiencing some form of discrimination or harassment, of which one case was reported to the awareness team.

### 2.8 How Do Perceptions of the Severity of EDI Issues Differ Among People With Different Social Identities?

To test for differences among social identities in their perceptions of EDI issues, we asked post‐congress respondents to indicate their agreement with the following three statements on a 7‐point Likert scale:

1. “I think the Congress attendees represented the diversity of researchers in our field” (“diversity represented” in short)
2. “Our research field experiences equity, diversity and inclusion‐related issues (e.g., racism, homophobia, harassment, bullying etc.)” (“EDI issues” in short)
3. “I think the questions asked after the talks were equally divided across genders” (“no QA gender disparity” in short).

We used the same analytical approach as described above for the congress experience models. However, instead of fitting “expertise rating” as an independent variable, we fitted age category, as we expected that older researchers would be more likely to have experienced different research environments as well as cultural diversity, and consequently, they might potentially have experienced more EDI issues independent of their level of expertise.

**FIGURE 6:**
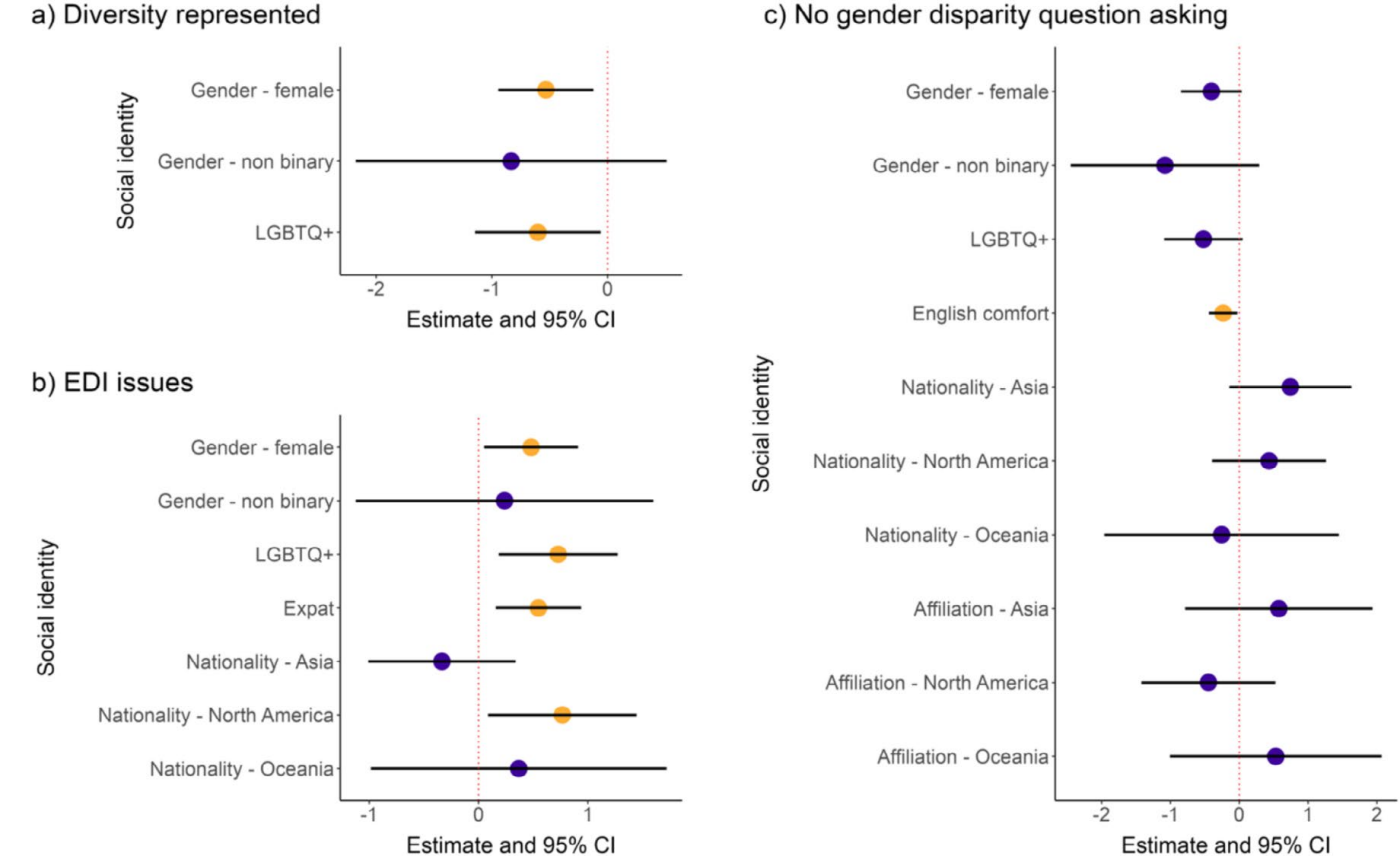
The results of models evaluating which social identities were significantly associated with variation in EDI issue perception. (a) Model estimates and 95% CIs of the final model that tested for the effect of social identity variables on the Likert‐scale response to the statement on congress attendees showing good representation of the diversity of the field. (b) Model estimates and 95% CIs of the final model that tested for the effect of social identity variables on the Likert‐scale response to the statement on our field experiencing EDI‐related issues. The reference continent for nationality to which the other continents were compared was Europe. (c) Model estimates and 95% CIs of the final model that tested for the effect of social identity variables on the Likert‐scale response to the statement on there being no gender disparity in question‐asking after talks. The reference continent for affiliation to which the other continents were compared to was Europe. The estimates and 95% CIs for African and South American nationalities and affiliations on statements (b) and (c) (Table S10) were excluded for easier visual presentation because the confidence intervals were large, which made visual interpretation of the other confidence intervals difficult. Yellow points indicate a statistically significant effect of the social identity variable in the final models.

Women agreed less with the “diversity represented” statement compared to men (*beta* estimate = −0.53, *p* = 0.01; Figure 6a; Table S10), and LGBTQ+ people agreed less to this statement compared to non‐LGBTQ+ people (*beta* estimate = −0.60, *p* = 0.03; Figure 6a; Table S10). Similarly, women agreed more with the “EDI issues” statement compared to men (ordinal *beta* estimate = 0.48, *p* = 0.03; Figure 6b; Table S10), and LGBTQ+ identities agreed more to this statement compared to non‐LGBTQ+ identities (*beta* estimate = 0.73, *p* = 0.009; Figure 6b; Table S10). Moreover, expats agreed more with the statement on EDI issues compared to non‐expats (*beta* estimate = 0.55, *p* = 0.006; Figure 6b; Table S10). Furthermore, compared to people of European nationalities, people with North American nationalities (*beta* estimate = 0.77, *p* = 0.03; Figure 6b; Table S10) agreed more with the “EDI issue” statement. Lastly, people of South American nationalities agreed more to the “no QA gender disparity” statement (ordinal *beta* estimate = 2.64, *p* = 0.04; Table S10) compared to people with European nationalities, although those with South American affiliations agreed less compared to those with European affiliations (ordinal *beta* estimate = −5.39, *p* = 0.006; Table S10), a contradicting result which could have arisen due to low sample size. People who are more comfortable speaking English agreed less with the statement about no QA gender disparity (ordinal *beta* estimate = −0.23, *p* = 0.03, Figure 6c; Table S10). Although including gender, LGBTQ+ identity and nationality significantly improved model fit in the univariate regression models for no QA gender disparity, they did not explain significant variation in the final model that included all significant covariates (Table S10). This means that when controlling for all other covariates, gender, LGBTQ+ identity and nationality did not explain any significant variation in the “no QA gender disparity” response.

### 2.9 What Can Be Done to Promote Inclusivity at Scientific Conferences?

The organising committee took a number of measures to make the Behaviour 2023 congress more inclusive. We asked participants to respond to an open‐ended question in the post‐congress survey to obtain qualitative feedback on, for example, the various inclusivity initiatives taken, overall participant experience, and suggestions for improvement. Of the 391 total respondents, 48% (*n* = 191) provided a response to this question, of which 185 could be assigned to a particular topic (i.e., a “code,” for details, see the Section 4).

**FIGURE 7:**
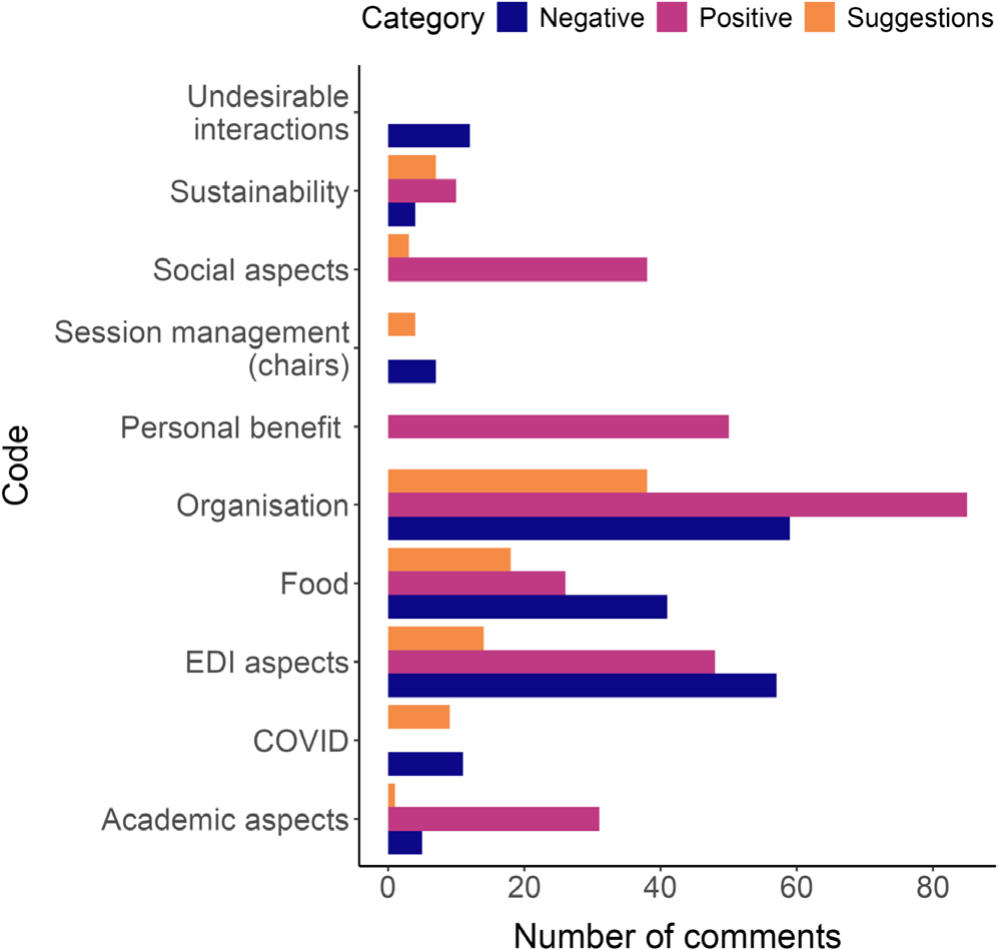
Frequency of ideas expressed in each category for the three sentiments (positive, negative, suggestion).

Most of the open‐ended responses in the post‐congress survey consisted of a combination of three sentiments (positive, suggestions, negative; Table S11); however, 51 responses contained only positive feedback, 22 contained only negative feedback, and 4 contained only suggestions. We coded 691 elements across 24 codes. Among these were 112 general compliments on the conference (e.g., “Great conference, thank you.”) that were excluded from further analyses. Of the remaining 579 elements, 50% (*n* = 288) were positive, 34% (*n* = 197) were negative, and 16% (*n* = 94) were suggestions (Figure 7). While the participants offered feedback on a number of different topics, multiple responses included feedback about one or more specific EDI‐related measures taken during the congress, including the positive impact our EDI initiatives had on their experience, which we elaborate on below. Although such feedback was relatively infrequent, we argue that this is as expected, as these measures are often only perceived by the ones who need them the most. We report these numbers as well as direct quotes from respondents to illustrate the positive impact that these measures can have, and our main take‐aways from the qualitative feedback can be found in Table 1.

1. *Plenary speaker diversity*. A few (<5) participants mentioned their appreciation for gender and/or ethnic diversity in plenary speakers, with one person indicating why this was appreciated, e.g., “It makes a huge difference to see gender and ethnic diversity represented in these headline names, so well done on selecting this set of speakers. It sets a positive tone for the whole meeting.”
2. *Pronouns on name tags*. A few (<5) people thanked us for allowing the option to print pronouns on their nametags (of which not all were non‐binary), where one person commented that they appreciated the option as they “care about making sure everyone can feel more included just by default”.
3. *Code of Conduct and awareness team*. The official Behaviour 2023 website contained a webpage on “Inclusivity and Accessibility” which included the Code of Conduct and additional information on who to contact about special needs. The responses from the post‐congress survey indicated that 43% of respondents read this webpage. Out of those that read the page, 25.6% of respondents indicated that it played a role in their decision to attend the congress. Although there is no direct evidence for this, this figure could indicate that the conference attracted people who value inclusivity initiatives. A total of 19 people mentioned in the open text that they appreciated our general push for inclusivity at the congress, with a few (<5) people specifically mentioning the Code of Conduct and/or awareness team. Some of them highlighted how the presence of the awareness team helped them feel safe, e.g., “I was very grateful that the awareness team existed, which really helped me feel safe during this conference”.
4. *Childcare*. A total of 11 people who filled out the survey used the free childcare service offered during the congress, seven of whom stated that they would not have been able to attend the congress without this service. Seven respondents also indicated that they would be able to attend more conferences if (free) childcare was available as a standard. The responses to the open‐ended question in the post‐congress survey included five positive mentions of the free childcare provided, where one person highlighted the difference this makes in the conference experience of parents, e.g., “After becoming a parent this was the first conference I could really enjoy fully and focus on the lectures and talking with colleagues.”
5. *Accessibility/disability*. A total of 18 people indicated in the post‐congress survey that they have some form of a disability, although 11 did not inform us about this prior to the conference. Out of those that did, three indicated that we were able to accommodate their disability, five indicated that the accommodation could have been better, and one person said that we were not able to accommodate their disability. The qualitative feedback included comments and suggestions for event organisers in general to make scientific conferences more accessible and inclusive, especially for researchers with a disability. The common themes of these comments included: (i) the difficulty of moving around the conference venue for people with mobility issues (in our case, mostly related to distances and stairs in the lecture rooms), (ii) the distraction caused by using (animal) sounds to indicate time limits to speakers, (iii) the appreciation of a quiet room for everyone who needs a space to “recharge and reflect”, (iv) the overwhelming experience during poster sessions that was non‐inclusive to people sensitive to sound and/or prone to anxiety in large crowds, and (v) the importance of ensuring the availability of presentation programmes' notes that can be seen by only the presenter during the talk.
6. *EDI‐related activities*. A total of 66 people that responded to the post‐congress survey attended the EDI symposium, and 21 attended one of the EDI workshops (one on unconscious bias and one on inclusive teaching). Reasons for attending the symposium and/or workshop included being motivated to (i) learn about EDI issues (61% and 66%, respectively), (ii) improve the way they do research (61% and 67%), and (iii) talk about their own (10% and 24%, respectively) or others' (18% and 62%, respectively) EDI‐related issues. Out of the reported symposium/workshop attendees, many respondents stated that attending will influence their practice, with some being sure about the changes they would make (41% and 29%, respectively), and others seeing the potential but being less sure (20% and 62%, respectively). Suggestions for EDI‐related workshops in general, which were not specific to the content and facilitators of the workshops we hosted in particular, mostly focused on the need to shift from theoretical work to practical implications.

**TABLE 1:** Summary of our recommendations for more inclusive scientific events (prior to (a) and during (b) the congress), our recommendations for more inclusive environments in general (c), and avenues for future research (d).

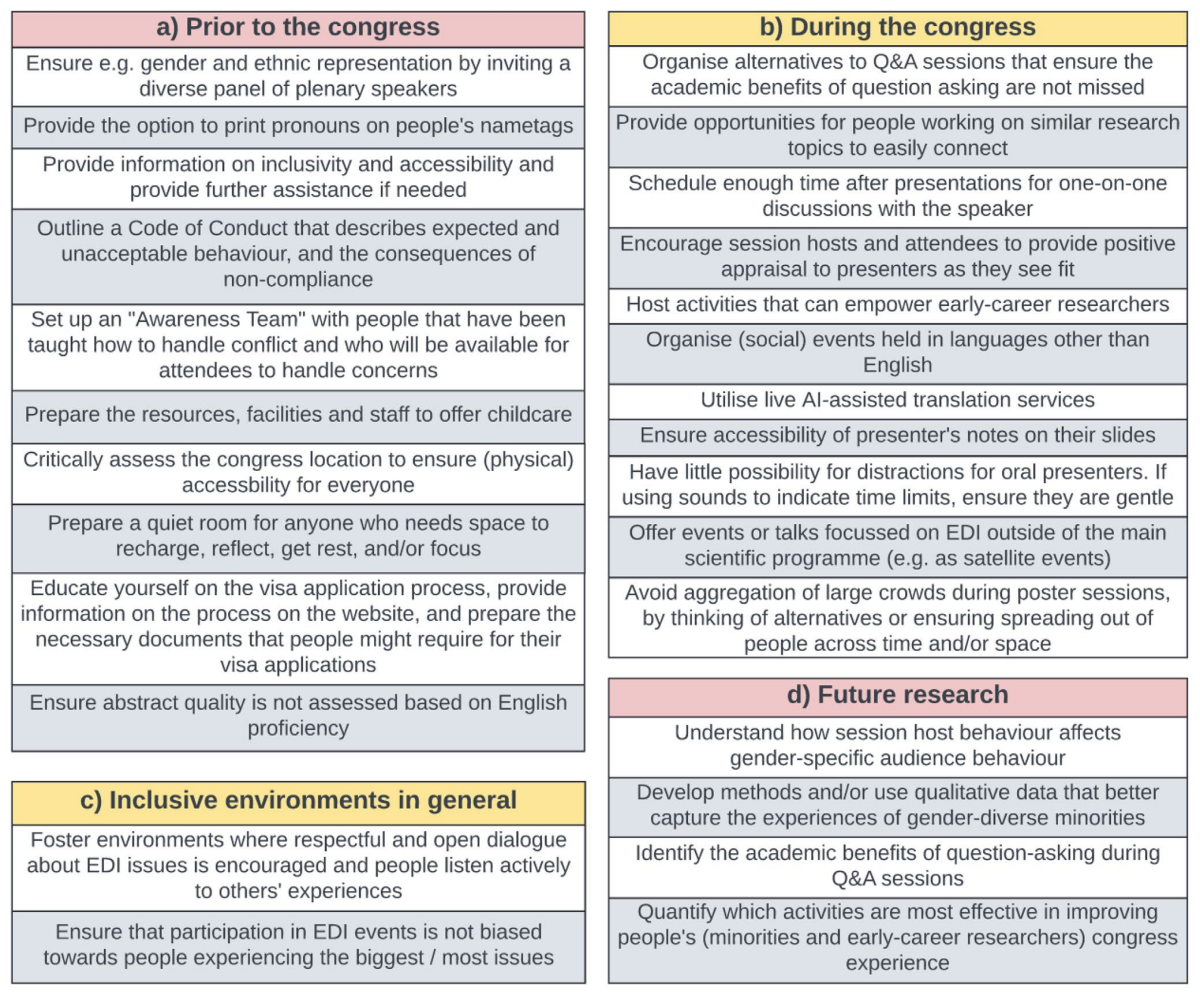

*Note:* Our recommendations are based on the data we collected as well as our personal experience.

## 3 Discussion

**FIGURE 8:**
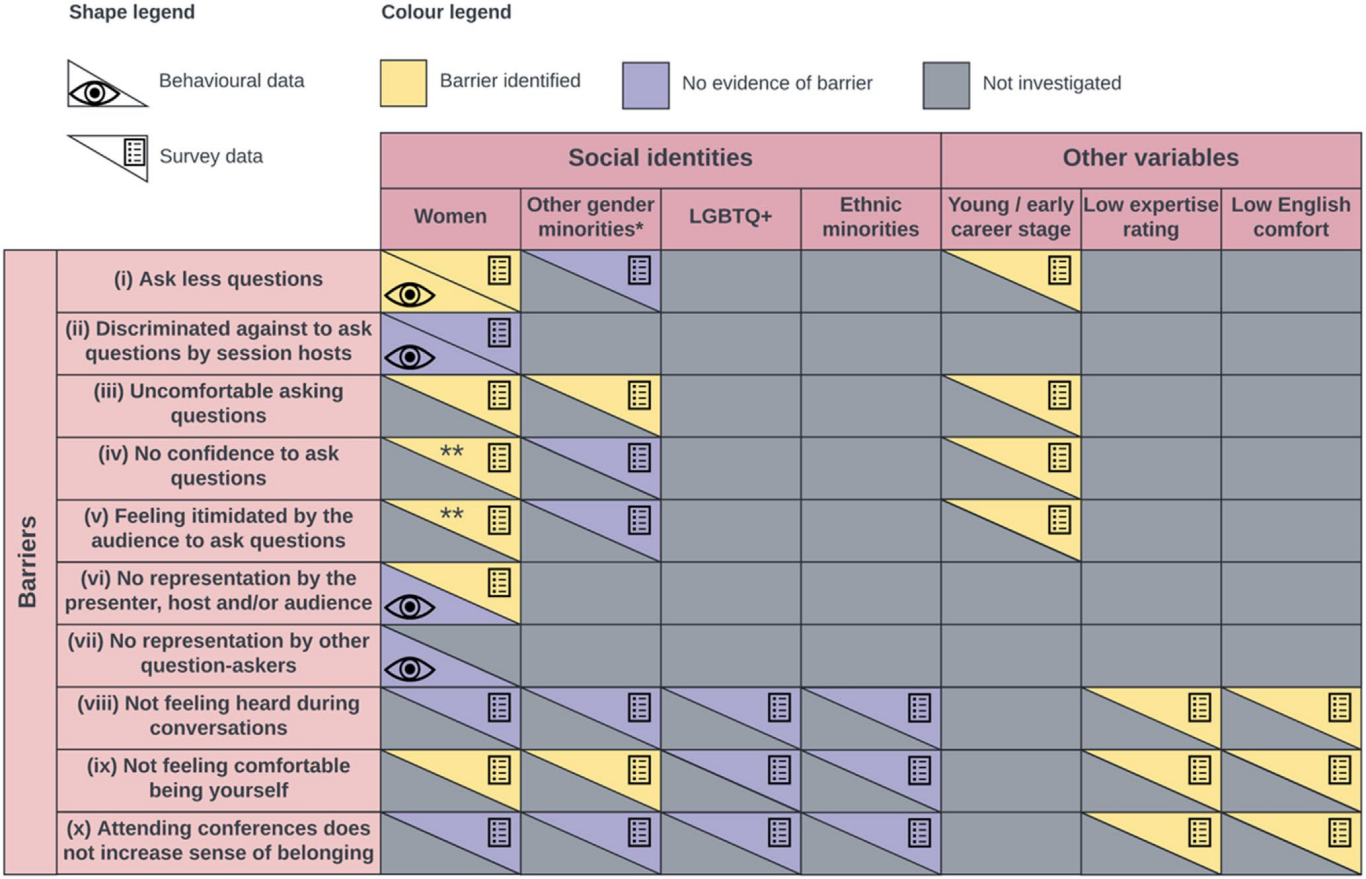
Summary of our results based on both the behavioural and survey data. The single asterisk (*) refers to non‐binary researchers. The double asterisk (**) refers to a marginally significant result (not significant after applying a multiple‐testing correction).

Barriers to inclusion and equity persist in science, including at academic conferences on behaviour, ecology and evolution. Our aim was to identify and address EDI issues present at the 37th International Ethological Congress that stretch beyond gender, using a number of different approaches. We identified barriers that unfold during Q&A sessions, as well as barriers that affect the congress experience of attendees not only when presenting or discussing science but also when simply attending the activities that are part of the conference programme. A summary of all results can be found in Figure 8, while all the recommendations we make for event organisers are highlighted in bold and can be found in Table 1.

### 3.1 Gender Disparities in Question‐Asking

We show that perceived women tend to ask fewer questions than perceived men, in line with research within and outside of biology (Carter et al. 2018; Hinsley et al. 2017; Pritchard et al. 2014; Schmidt and Davenport 2017), despite the fact that they do not appear to be chosen less often to ask their question by the session host. Although we find clear evidence that a question is less likely to be asked by a perceived woman compared to a man based on the behavioural data, self‐identified women only appear to be slightly less likely to have asked a question across the entire congress. This pattern may arise if men on average asked more questions per individual (e.g., three questions during the congress) compared to women (e.g., one question during the congress), which does not affect the probability that a woman asked a question in the survey but does affect the probability that a question was asked by a woman in the observational data. Alternatively, the pattern may arise if there are certain men that ask a lot of questions across different sessions or if women who did not ask any questions during the congress were also less likely to fill in our post‐congress survey.

### 3.2 Potential Reasons Why Women Ask Less Questions Than Men

We further found that self‐identified women likely ask fewer questions due to a lack of self‐confidence and because they feel intimidated by the audience (although only significant before applying a multiple‐testing correction). Indeed, the gender gap in confidence (Carlin et al. 2018; Vajapey et al. 2020), as well as the inaccuracy of women's self‐perception (Herbst 2020), has previously been proposed to play a role in various gender disparities, including the underrepresentation of women in senior leadership positions (Carlin et al. 2018). The reasons why women tend to have lower self‐confidence and belief in their own abilities are, however, complex and difficult to generalise, as they could be rooted in both internal and external processes that take place within and outside of the academic environment (e.g., family environment (Krauss et al. 2020), gender stereotypes (Master 2021), and a lack of role models (González‐Pérez et al. 2020)).

### 3.3 Women's Representation Did Not Always Encourage Women to Ask More Questions

Women's representation could potentially improve women's confidence, as it has been shown to boost female engagement (Bailey et al. 2020; Carter et al. 2018; Davenport et al. 2014; Hinsley et al. 2017), yet our findings only partially support this. Whereas the data collected in the post‐congress survey suggests that self‐identified women are more comfortable asking questions when their gender is represented (in the audience, by the presenter or session host), the data collected during the Q&A show that perceived women were less likely to raise their hand and ask questions than men, regardless of the situation. Moreover, perceived women appeared to be less inclined to raise their hands to ask questions, specifically after a woman started the Q&A. The exact mechanism behind this observation is unclear. One possible explanation is that women may be motivated to ask questions to ensure their gender is represented among the questioners. However, once another woman has already asked a question, others might no longer feel the motivation to ask a question.

While our results suggest that session hosts cannot mitigate the gender disparity in question‐asking by actively selecting women to start the Q&A, we found different results for the manipulated talks compared to the unmanipulated ones when a man started the Q&A. When host behaviour was not manipulated, we found no gender disparity when a man started the Q&A, as women were equally as likely to raise their hands. Yet, in our experiment, we did find a gender disparity in raising hands when a man started the Q&A. These results indicate that either the deliberate choice of a man over a woman (as happened in our manipulated talks) or the (conscious or unconscious) change in behaviour of the session host due to higher awareness of their choices might have discouraged women from asking questions during the rest of the session. Testing what exact perceived behaviours from session hosts affect the probability that women raise their hands to ask questions would require further research, yet the effects of female representation among questioners are evidently complex and appear to not always be positive.

### 3.4 Addressing Academic Opportunities Beyond Question‐Asking

While gaining a deeper understanding of the causes and consequences of gender disparities in question‐asking probability is important, we argue that it is more critical to ensure that women do not miss out on academic opportunities as a consequence of this disparity. The same accounts for non‐binary participants, who also appeared to be uncomfortable asking questions despite the limited sample size, although we require better methods and/or comprehensive qualitative data to capture the full experience of gender‐diverse minorities who cannot be identified unless their gender identity is disclosed. Questioners might gain academic benefits by (i) expressing their interest and participating in the scientific discussion, (ii) increasing their likability by showing responsiveness (Huang et al. 2017), (iii) growing their visibility, which can help them connect with people working on similar topics, and (iv) facilitating collaborations and/or exchanging ideas that can improve the quality of their research. To our knowledge, there is no empirical evidence of the academic benefits of question‐asking during Q&A sessions.

Assuming that there are benefits to question‐asking at conferences, we expect that similar outcomes could be achieved in alternative ways that might be more likely to be adopted by people who are less likely to ask questions, including but not limited to women. For example, conference organisers could plan topic‐focused discussion rounds, provide an online platform where attendees can connect based on mutual interests, and/or schedule more time after presentations for the audience members to engage in one‐on‐one discussions with the speaker. Such activities would benefit not only women but also introverted people and non‐native English speakers, who are less inclined to ask questions in Q&A sessions, as revealed by our quantitative and qualitative data. We thus urge a shift in focus towards addressing those potentially missed academic opportunities for people who are less inclined to ask questions during Q&A's, which disproportionately include women, and ensuring equity by providing alternative pathways to reclaim those opportunities.

### 3.5 Creating Positive Congress Experiences for Everyone

Moreover, our results have important implications with regard to differences in congress experiences. We found that women and non‐binary participants felt less comfortable being themselves, similar to philosophers identifying as women and non‐binary who feel less comfortable in professional and social settings (Jennings et al. 2019). Moreover, people who do not feel like an expert in the field, who are commonly more junior and/or younger researchers, appear to have a less positive congress experience. The qualitative data from the survey included statements indicating that critical questions from senior researchers can have a large negative effect on younger presenters, although we do not have data to test this relationship empirically. We do, however, suspect that the opposite is also true: senior researchers can have a positive influence on the experience of early‐career researchers through their feedback on oral presentations as well as during scientific discussions. Therefore, we encourage senior researchers to give positive appraisals to presenters when they see fit, which we expect to boost the congress experience by “warming up” the “chilly conference climate” that early‐career researchers might experience.

In addition, early‐career researchers should be empowered during conferences, which could be achieved by organising more specific events. For example, the organisational committee could (i) arrange Q&A sessions between (PhD) students and senior scientists, (ii) host events tailored towards early‐career researchers specifically, or (iii) set up a buddy network that connects (PhD) students who work on similar topics. Future research is, however, required to quantify which activities are most effective in improving the congress experience of younger and/or more junior researchers.

### 3.6 The Disadvantages of Being a Non‐Native English Speaker

Similarly, people who feel less comfortable speaking English also had a less positive congress experience. The dominance of the English language at international academic events causes a systemic bias. Indeed, recent work has started to uncover the many disadvantages faced by non‐native English speakers in academia (Amano et al. 2023). We encourage critical thinking about initiatives that can improve the inclusivity of people who are less comfortable speaking English, such as (i) hosting social events that accommodate foreign languages, for example language‐specific discussion rounds (also previously suggested by (Stefanoudis et al. 2021) and (Joo et al. 2022)), (ii) utilising AI‐assisted translation services during talks and/or Q&A sessions, similar to AI‐assisted academic writing (Giglio and Costa 2023; Hwang et al. 2023); (iii) allowing for abstract submissions in multiple languages (Joo et al. 2022) or alternatively, ensure that the assessment of the abstract quality is not based on English proficiency but on research quality only; and (iv) emphasising the importance of teaching English proficiency during early and higher education. Such activities have the potential to make people less comfortable speaking English feel more like they are heard, which can increase their sense of professional worth and belonging.

### 3.7 Recognition of EDI Issues Is Not Ubiquitous

Our results further show that different social identities have dissimilar perceptions of EDI issues. Evidently, historically underrepresented minorities, including women and LGBTQ+ identities, seem to better recognise EDI issues. Previous research has also shown that men are less likely to notice gender disparities in question‐asking probability (Lupon et al. 2021). We expect that minorities are more likely to notice EDI issues either because these groups experience more EDI issues themselves or because they are more aware of issues that other people face, or a combination of the above. Interestingly, expat scientists agree more with the statement that our field (behavioural, ecological and evolutionary sciences) experiences EDI issues. Although there can be culturally ignorant expats and culturally aware non‐expats, our strong significant result despite this potential noise highlights that overall, there seems to be a link between expatriation and cultural intelligence (Morin and Talbot 2023). Our findings emphasise the importance of active listening (Decady Guijarro and Bourgeault 2023), especially to those with a cultural background or social identity different from one's own, which can increase awareness of issues both inside and outside of academia.

The importance and value of listening are directly reflected by the comprehensive constructive feedback that we received in the post‐congress survey. Many congress attendees took the opportunity to provide suggestions for making conferences more inclusive and raised both minor and major points for improvement that would not have been brought to our attention if we had not specifically asked for this feedback. We therefore encourage every research group to provide the opportunity for members to express their concerns and to foster an environment where dialogue about EDI issues is encouraged (Holmes et al. 2016), while ensuring that the participation in these conversations is not biased toward marginalised people only.

### 3.8 Small Actions Can Boost Inclusivity at Scientific Conferences

The responses to the open‐ended questions in the post‐congress survey revealed that participants had an overall positive experience during the conference. Nonetheless, there were also critiques and suggestions that were not only specific to this event but could be relevant to scientific conferences in general. Although we are aware of the many logistic, financial, and time‐related limitations that event organisers face, we would like to emphasise a number of aspects that have been suggested by respondents to foster more inclusive conferences. We think these aspects can be addressed to improve the experience of the minority without sacrificing the experience of the majority by making small tweaks or implementing small additions to accommodate everyone.

First, giving an oral presentation can itself be stressful regardless of a person's social identity and abilities. Attention to a few simple details can help mitigate some of this stress. For example, ensuring that the presentation programme's notes are available to the presenter can especially benefit neurodivergent and non‐native language speakers. Stress can additionally be lowered by limiting the scope for distractions, such as auditory cues indicating the presentation time remaining. Although these cues can be helpful for the majority of people, if they are played too loud, they can be distracting to neurodivergent speakers with heightened auditory sensitivity. So, we encourage event organisers to ensure such sounds are played at an appropriate volume for everyone.

Secondly, although international conferences in theory provide an excellent opportunity to host workshops on EDI‐related themes, we believe that such workshops are likely to be more effective if they are organised as satellite events. This way, the workshops can be longer in duration, allowing for the discussion of both theoretical and practical aspects; attendees do not have to choose between attending workshops or scientific talks, and having these satellite events during the year can help increase interactions and build community.

Lastly, poster sessions held in loud, crowded venues can be overwhelming, especially for people sensitive to large crowds and/or auditory overstimulation. Alternatives to poster sessions have previously been proposed (e.g., virtual posters (Arcila Hernández et al. 2022; Holt et al. 2020)), and we encourage future event organisers to critically think about the setup, size and location of the poster sessions and/or alternative modes for more inclusive and equitable ways of presenting science. This does not necessarily have to go at the expense of traditional poster sessions, which are effective for the majority of attendees, but we encourage having alternative options available.

### 3.9 The Statistical Limitations of Analysing Minority Groups

Several inferences about certain groups of social identities made in our study are based on relatively low sample sizes. We acknowledge the statistical limitations of these inferences; nevertheless, we argue that these inferences address barriers experienced by social minorities that have rarely been researched. For example, we find a clear signal that non‐binary respondents felt uncomfortable being themselves in the post‐congress survey even though there were only seven non‐binary respondents. Including this small group of people in our analysis helps to illuminate the social barriers faced by certain minorities, which by definition are represented in small numbers. We further argue that, as opposed to quantitative analyses, qualitative data can be more insightful in identifying and addressing barriers experienced by minorities, as shown by the comprehensive feedback given by the handful of respondents on mobility‐ and neurodiversity‐related issues. Qualitative data can furthermore help shine light on intersectional inclusivity issues.

### 3.10 Broader Implications for Academic Settings

Our case study investigated equity, diversity, and inclusivity issues at an academic conference hosted in Europe. Although our conclusions are mainly drawn based on a European audience, which could induce a bias, we expect that many of the inferences that we draw from our data collected at this large, international conference can be generalised to academic conferences in general but also settings outside of conferences focused on biological sciences. For example, our conclusions on question‐asking behaviour are likely to be applicable to Q&A sessions not only at conferences but also within the setting of seminars given at academic institutes. We also expect that our findings on differences in congress experiences between people of different genders, with different levels of comfort in speaking English, and with different perceived levels of expertise will be applicable to many different academic social settings, such as lab meetings and collaborative projects. Our study therefore does not only have implications for the way we host and attend scientific events, including conferences, but also for conducting science overall. Removing barriers that are present across different academic settings requires acknowledgement of those barriers, especially by those in leadership positions, identifying the causes and mechanisms by which these barriers are established and maintained, understanding how they affect researchers, and developing effective strategies to tackle them through open, accepting, and respectful dialogue.

## 4 Methods

### 4.1 Conference Description

Bielefeld University, located in Germany, hosted a seven‐day International Ethological Congress, “Behaviour 2023,” in August 2023, which was attended by 855 people. The official language of the congress was English. Six of the days consisted of scientific talks, including 11 plenary talks given by invited international speakers, which lasted 60 min each, including a 10–15‐min question‐and‐answer (Q&A) session. After each plenary talk (except on the last day), oral sessions took place, which consisted of one to seven talks. In total, there were 56 general oral sessions, as well as 42 oral sessions that were part of symposia on a specific theme. General oral sessions and symposia were moderated by internal and/or external session hosts. Each talk slot lasted 15 min, with the speakers being instructed to limit their speaking time to a duration of 12 min, leaving three minutes for the Q&A. Each day (except the last day) consisted of parallel morning and afternoon sessions, and each session included a coffee break.

Various initiatives were taken to promote inclusivity at Behaviour 2023. First, all of the congress attendees were obliged to agree to a Code of Conduct when registering for the congress. The Code of Conduct outlined expected and unacceptable behaviours and clearly stated the consequences of non‐compliance. During the congress, attendees were able to inform an awareness team about any concerns and cases of discrimination or harassment. The awareness team was a group of organising committee members who had received harassment training from an external organisation (Frauen Notruf Bielefeld e.V.) who could be contacted by email, phone, via social media, or directly in person during the congress. Recognition of awareness team members was facilitated by them wearing a recognisable badge.

Moreover, the programme of plenary talks was curated in a way that ensured a balanced representation of gender and ethnic diversity among plenary speakers, ensuring that at least half of the plenary speakers were female and that each continent was represented at least once. Prior to the congress, we offered information and help to people with auditory, visual, mobility and/or dietary needs through the website and during congress registration. We offered a number of full travel grants to researchers based in the Global South. During the congress, we offered free childcare provided by an external company, which was funded by the Bielefeld Equal Opportunities Committee. We additionally offered parent‐children offices, breastfeeding rooms and free congress attendance to the partners of attendees that were only there to provide childcare. We further offered quiet rooms that were open between at least the first and last talk of each day. Moreover, we convened a symposium on “Equality, diversity and equity in behaviour, ecology and evolution” with talks given by three invited speakers and organised three half‐day workshops given by external moderators in an attempt to foster engagement and critical dialogue on EDI issues among congress attendees. We organised workshops on two different topics: one on unconscious bias and one on inclusive teaching in higher education. The former workshop was given two times on the same day, independently from each other with different groups of workshop attendees. Lastly, we offered the option to congress attendees to print their pronouns on their nametags, in an attempt to avoid misgendering among congress attendees and to build an inclusive culture for non‐binary people.

### 4.2 Pre‐Congress Survey

Congress attendees were asked to fill in a voluntary online survey as part of the online congress registration on their social identity. The survey included questions on (i) their pronouns, (ii) if they identified as lesbian, gay, bisexual, transgender, queer, intersex or any other non‐heterosexual, non‐heteroromantic, or non‐cisgender identity (LGBTQ+), (iii) their nationality, and (iv) if they have any disabilities. As not all congress attendees registered through the online portal, and not all attendees filled in the voluntary online survey, the social identity information of congress attendees is not 100% complete.

### 4.3 Question‐Asking Study

We collected data on question‐asking behaviour during Q&A sessions at the congress. Although it is important to understand disparities in question‐asking behaviour among multiple social identities as well as the intersections of those identities, we focused only on gender disparities due to logistical and practical reasons, as this was the most conspicuous identity that could be perceived in a real‐life setting. We observed the question‐asking behaviour of the participants of 67 oral sessions at the congress. No informed consent for this part of our study was obtained, as the study was anonymous and conducted in a public space, and notifying congress attendees about our study and its intentions could have biased their behaviour, as previously noted by Hinsley et al. (2017). All methods used in our study were approved by the Ethical Committee of Bielefeld University.

A total of 25 observers (organising committee members, students and/or colleagues working in biology) collected data on question‐asking behaviour across the five days of talks. 62.5% of observers were women, and 37.5% were men (no non‐binary identities); they represented 14 nationalities, and 16.7% identified as LGBTQI+. All observers were made aware of the spectrum of gender diversity and the complexities of perceiving gender during comprehensive internal training sessions and discussions. Observers were randomly allocated to collect data in oral sessions within the timeframe of their availability. When collecting data, observers conducting the study were seated in the back corner(s) of the lecture hall to obtain a better overview of the audience and to reduce our visibility when counting the number of people in the audience (see below). In 32 of the 67 sampled sessions (48%), data were gathered by multiple observers to evaluate inter‐observer reliability (hereafter referred to as “double‐sampled sessions”). Sessions were held in lecture halls of three different sizes: small (63–77 seats), medium (102–132 seats) and large (308–404 seats). Because it is difficult to observe people in large lecture rooms while remaining stationary, sessions held in large rooms were always sampled by two observers, where some variables were collected by one observer but not the other and vice versa (see below). Therefore, data collection in a double‐sampled session in a large room was done by four people.

We collected data on the perceived gender (woman, man, other) of session hosts, speakers, and questioners (see below). Data were collected at three different levels: per session, per talk, and per question, as described below.

#### 4.3.1 Data Collected Per Oral Session

For each oral session, we noted down the perceived gender of the session host, as well as three meta‐data variables, including the day of the congress (day 1–5), lecture hall (1–9), and whether the session was part of a general oral session or symposium. Although general oral sessions were hosted by just one person, a symposium could be hosted by up to three session hosts. If a symposium was hosted by more than one person, we focused on the host that led the Q&A session. If multiple hosts led the Q&A session, or if the hosts swapped roles, this was noted down and accounted for in the relevant analyses as described below.

#### 4.3.2 Data Collected Per Talk

At the start of each talk, the total audience size was counted, as well as the total number of perceived men in the audience. These data allowed us to correct for the audience gender proportion on a talk‐by‐talk level. The session hosts of the focal session, the current speaker, observers and technical assistants were excluded from these counts, as the gender of the current speaker is analysed separately, and observers and technical assistants did not ask questions. We noted down if there was any uncertainty in the number of people counted due to, for example, the view of the observer being partially blocked, people sitting in areas out of sight of the observer, or limited light in the room. We recorded the perceived gender of the speaker, the duration of the Q&A session in minutes, and noted occasions when the speaker talked for longer than their allocated time slot.

#### 4.3.3 Data Collected Per Question

For each question asked after each talk, we counted the total number of people and the total number of perceived men who raised their hands to ask a question. Because it was more difficult to reliably count all of the people who raised their hands in large rooms, two observers were always present in the large rooms (and four people in double‐sampled large rooms). One of the two observers counted the total number of people raising their hands and the other observer counted only the number of perceived men who raised their hands. For each person who asked a question, the following data were collected: the perceived gender of the person asking the question, if they showed appreciation towards the speaker (e.g., “Thank you for the interesting talk”) and whether the question contained criticism and/or a counterargument. Lastly, the observers noted down if one of the following situations occurred: a person asked a question without raising their hand (“jumper”), the session host asked the question, the speaker chose who asked the question instead of the session host, a person asked multiple questions in one turn, or a person asked multiple questions in one Q&A but not consecutively.

#### 4.3.4 Data Collected During Plenary Talks

Plenary talks were held in a different building with a large lecture hall containing 638 seats and were not run in parallel with any of the other congress activities. Due to the difficulty of counting the number of people sitting down and raising their hands in this large lecture room, we only collected data on the perceived gender of the people asking questions. At least two observers collected data during plenary talks, and the perceived gender and number of questions for plenary talks were manually cross‐checked based on the notes taken by each observer.

#### 4.3.5 Perceiving Gender

The gender of session hosts, speakers, and questioners was perceived using a combination of numerous sources of information: pronouns (printed on nametags and/or mentions during introductory slides), visual cues (hair length, clothing, body size, facial features), auditory cues (voice pitch), and a person's name if stated when asking the question. These sources of information are central to social cognition and are consciously and unconsciously used to categorise genders in social settings (Brown and Perrett 1993; Pernet and Belin 2012; Weißflog and Grigoryan 2024), a process that is largely automatic (Tomelleri and Castelli 2012). There are, however, limits to perceiving gender based on a person's appearance, which are particularly pronounced for people falling outside of or between the men–women gender binary. This is because gender‐ambiguous targets are complex to categorise (Weißflog and Grigoryan 2024), and even if pronouns of the person are known, they are not always indicative of gender identity (Moeder et al. 2024). Moreover, non‐binary identities can be blended or concealed depending on the person (Flynn and Smith 2021) and are therefore difficult to perceive unless deliberately disclosed. We therefore only evaluate the experience of explicitly disclosed non‐binary identities during Q&A sessions using the post‐congress survey (see below).

#### 4.3.6 Experimental Manipulation of Session Host Choice

We investigated if the session host's choice of questioner can help overcome gender disparity in question‐asking probability. For a subset of sessions (40 sessions, 62.5%), we manipulated the behaviour of the session host. We used stratified random assignment of session hosts to either an unmanipulated or manipulated session. If the session host was part of the organising committee, they were automatically assigned to a manipulated session because they were aware of the study and its purposes, and consequently, they might be biased if assigned to an unmanipulated session. The hosts of unmanipulated sessions were unaware of our study and were not contacted prior to the congress about the study to ensure their behaviour was ‘natural’ and unbiased by our study intentions. Two weeks prior to the congress, the hosts of manipulated sessions were asked by email if they wanted to participate in our study, without mentioning the exact goal or describing the tasks in detail. If the session host agreed, they were given instructions specific to their session. If the session host declined to participate (*n* = 2), we did not sample that session and swapped data collection with a session whose host agreed to participate.

In manipulated sessions, the host was instructed for each talk within that session to assign the first question of the Q&A to either a woman or a man, resulting in two possible conditions. The conditions were randomly assigned across all of the talks in all of the manipulated sessions, ensuring an overall equal distribution of the two conditions over all sampled talks but not necessarily an equal distribution of the two conditions within a manipulated session. If the raising of hands did not meet the experimental condition (e.g., the condition was the first question given to a woman, but no women raised their hands), the hosts were instructed to select a person as they normally would.

Hosts successfully assigned the first question to the assigned gender in 102 talks (48 to a perceived woman and 54 to a perceived man). The manipulation was unsuccessful in 106 talks, either because nobody of the assigned perceived gender raised their hand (*n* = 63) or because of other unknown reasons (*n* = 43).

#### 4.3.7 Data Curation and Validation

A number of steps were taken to curate the collected data on question‐asking into the final dataset used for analyses, which are described in detail in Appendix A. Briefly, we checked whether data collected in double‐sampled sessions had a good inter‐observer reliability. Indeed, agreement between observers was “good” to “almost perfect” for all of the variables (Appendix B).

Because there were slight differences in how certain situations were noted down by observers of double‐sampled sessions, we manually checked and corrected the data when the observers appeared to disagree over the number of questions that were asked (9 talks). After manual correction, data from different observers of the same session were combined using a conditional workflow dependent on the variable as described in Appendix C. Briefly, (i) if observers disagreed on the perceived gender of a person, we discarded the data, as this might mean that observer perception is not indicative of audience perception of a person's gender; (ii) we took the mean of audience number estimations; (iii) we used the maximum of the number of hands raised, and (iv) we assumed that disagreement on the variables that recorded whether something was or was not done or said (e.g., a questioner appreciating the speaker) was due to one observer having missed it or forgetting to note it down rather than the other observer taking note of something that did not happen or was not said.

#### 4.3.8 Statistical Analyses of Behavioural Data on Question‐Asking

To test whether there was a gender disparity in question‐asking probability, we built a series of generalised linear mixed effect models (GLMMs) using the R package lme4 v1.1 (Bates et al. 2015). Unless indicated otherwise, the data used to construct the models below excluded sessions where we manipulated session host behaviour, as well as questions that were follow‐up questions by the same person, questions asked by the session host, or questions asked by people who did not raise their hands (jumpers). For clarity, a summary of the models that use the observational data can be found in Table S12, which includes a clarification of the subset of the data used, the research question it addresses, and the formula written in lme4 syntax (Bates et al. 2015).

The first model (QA.1) tested whether perceived women ask fewer questions than perceived men do in regular oral sessions. We fitted a binomial GLMM to the perceived gender of the questioner (1 = female, 0 = male). Under the null hypothesis, we would expect that the proportion of questions asked by women is equal to the proportion of women in the audience. This would therefore mean that the audience consists of 60% women; the null hypothesis is that 60% of questions are asked by women. Therefore, we corrected for the gender proportion of the audience by specifying the *offset* argument in the GLMM as the logit of the proportion of women in the audience. We corrected for the non‐independence of talks within a session by including the random effect of talk ID nested within session ID. If the resulting intercept was significantly negative, this would indicate that women asked fewer questions than men did. We repeated this analysis with a conservative subset of the data that excluded any questions where there was uncertainty in the data, for example, because the observer could not count the audience reliably or if the gender of a person was ambiguous (QA.1c).

We also tested for gender disparity in question‐asking probability in the plenary sessions only. A similar GLMM was fitted as described above (QA.1) using the observational data collected during plenary talks, where the dependent variable was the perceived gender of the questioner and a random effect was included for plenary ID (QA.1p). Because of the large audience and room size, it was not possible to accurately count the number of perceived women and perceived men in the audience. Therefore, instead of correcting for the proportion of the perceived women in the audience, we corrected for the gender proportion by using the proportion of congress registrations who used she/her pronouns, which should be indicative of the proportion of registrations identifying as women (REF). This method assumes that the vast majority of registrants attended the plenary sessions.

Next, we used a similar model structure to model QA.1 to address what conditions can encourage perceived women to ask questions. Specifically, we tested for the effects of the following five variables on the gender disparity in question‐asking probability: (a) the perceived gender of the speaker (male, female or non‐binary), (b) the perceived gender proportion of the audience (where 1 would theoretically indicate an audience consisting of 100% perceived women), (c) the perceived gender of the session host (man, woman or other/non‐binary), (d) the total size of the audience, and (e) the size of the room (small, medium or large), further referred to as models QA.1a—QA.1e, respectively. We constructed five binomial GLMMs using the inferred gender of the questioner as the dependent variable and one of the five variables as an independent variable. We again corrected for the gender proportion of the audience using the *offset* function as described above and included the random effect of talk ID nested within session ID. For the model that tests for the gender of the session host (QA.1c), we excluded sessions where there were multiple session hosts who alternated leading the Q&A. We determined whether a variable was a significant predictor of the likelihood that a woman asked a question by conducting a likelihood‐ratio test (LRT) using the *anova* function from the stats R package v4.3.2 (Team 2021), which compared the model in question with the null model that only included the intercept (QA.1).

#### 4.3.9 How Does a Gender Bias in Question‐Asking Arise?

Perceived women might ask fewer questions than men do due to two different reasons: perceived women raise their hands less often than men do, or perceived women are chosen less often to ask their question by session hosts when they do raise their hands. We tested which reason was the most probable cause for the gender disparity in question‐asking probability by fitting two GLMMs.

The first GLMM (QA.2) evaluated whether perceived women raised their hands less often than perceived men did by fitting the number of hands raised by perceived women and men as the response variable using the *cbind* function. Similar to above, we corrected for the gender proportion of the audience by specifying the *offset* argument as the logit of the proportion of women in the audience. Again, we used a binomial error distribution and corrected for the non‐independence of talks within a session by including the random effect of talk ID within session ID. Under the null hypothesis, we expected that the number of hands raised by women and men would be proportional to the number of female and male audience members, respectively. If the resulting intercept was significantly negative, this would indicate that women raised their hands less often than men did.

The second GLMM (QA.3) evaluated whether perceived women were chosen less often by session hosts than perceived men were by fitting the perceived gender of the questioner as the response variable, but instead of correcting for the perceived gender proportion of the audience, we corrected for the proportion of perceived women out of those people who raised their hands. Under the null hypothesis, we expected that the number of questions asked by women would be proportional to the number of women who raised their hand. We therefore specified the *offset* argument as the logit of the proportion of women out of the people who raised their hands. For this analysis, we only used a subset of the data where the session host could make a choice between allocating the question to a man or woman, meaning that the subset only included situations where at least one woman and one man raised their hand. We again used a binomial error distribution and corrected for the non‐independence of talks within a session by including the random effect of talk ID within session ID. If the resulting intercept was significantly negative, this would indicate that women were chosen less often to ask their question than men were.

#### 4.3.10 Do Women Ask More Questions if Other Women Have Asked Questions Previously in the Q&A?

Session hosts can potentially help to reduce the gender disparity in question‐asking probability by selecting women to ask the first question and/or by encouraging other women to raise their hands and ask questions. We tested whether the perceived gender of the first questioner affected the probability of (i) a perceived woman asking a question compared to the proportion of perceived women in the audience, (ii) a perceived woman raising their hand and (iii) a perceived woman being chosen to ask their question compared to the proportion of people raising their hand who are perceived women by fitting three different binomial GLMMs to unmanipulated talks only. We used similar models to QA.1 (the response was the gender of the questioner, corrected for the gender proportion of the audience), QA.2 (the response was the gender of the people who raised their hands, corrected for the gender proportion of the audience), and QA.3 (the response was the gender of the questioner, corrected for the proportion of women out of the people who raised their hands), respectively. Additionally, we excluded the first question asked in each Q&A session from the dataset and used the gender of the first questioner as a fixed effect instead, as the gender of this first questioner was our variable of interest. We removed the intercept (by adding −1 to the formula) to allow for an easier interpretation of the output. For clarity, a summary of the models that address the effect of the gender of the first questioner can be found in Table S13, which includes a clarification of the subset of the data used, the research question it addresses, and the formula written in lme4 syntax (Bates et al. 2015).

The three models were fitted using two separate datasets, first using the data collected in unmanipulated sessions only (QA.4u–QA6u, respectively) and second using data collected in manipulated sessions where the first question was successfully assigned according to the condition of the manipulation (i.e., a woman or man asked the first question as instructed, QA.4m–6m, respectively). We repeated all six GLMMs with a subset of the data that only included the second question asked in each session (QA.4u.2–QA.46u.2 and QA.4m.2–QA.46m.2, respectively) rather than all questions asked after the first one. These models helped us determine whether the gender of the first questioner only affected the probability that only the next question was asked by a woman, rather than all questions in the remainder of the session. To test whether the gender of the first questioner had a significant effect on the response variable, we compared the fit of the model to a null model that only included the random factors using an LRT.

All of the models described above excluded follow‐up questions by the same questioner, cases where the speaker assigned the question rather than the host, questions asked by the session host, questions asked by jumpers, and questions where the gender of the questioner or the proportion of women in the audience was unknown. The models using the number of hands raised (QA.2, QA.3, QA.5u, QA.5m, QA.6u, QA.6m) also excluded cases where the number of women and/or men raising their hands was unrecorded (e.g., because the observer did not see it) or when no hands were raised. The models where the probability of being chosen to ask a question was investigated (QA.3, QA.6m, QA.6u) excluded cases where only men or only women raised their hands, as here the host could not choose whether a woman or man got to ask their question. The model estimates (predicted log‐odds) were obtained from Wald tests using the *summary* function in lme4 v1.1‐35.5 and back‐transformed to probabilities (inverse logit) using the *plogis* function in stats v4.4. We additionally obtained profiled confidence intervals using the *confint* function in stats v4.4. A probability was considered to be significantly different from the expected probability under the null hypothesis (no gender disparity, probability = 0.5) if the *p‐*value of the Wald test was lower than 0.05 and if the corresponding 95% confidence intervals did not overlap zero.

#### 4.3.11 Other Gender Disparities in Oral Sessions

We further investigated whether men or women have a higher probability to: (i) ask a question without being chosen to (i.e., being a “jumper”), (ii) speak for longer than their allocated time, (iii) give and/or receive a compliment after an oral presentation, and (iv) ask and/or receive critical questions. We investigated which variables of interest (e.g., gender) were significantly associated with the probability that one of the four mentioned cases occurred by constructing a binomial GLMM for each of the dependent variables of interest (Table S14). Statistical significance of the variable was inferred from an LRT which determined whether including the variable significantly improved the fit of the model compared to the null model that did not include the variable. Only statistically significant predictors (LRT *p*‐value < 0.05) were retained in the final model. In all of these models, we included the random effect of talk ID nested in session ID. The results of these models are described in Appendix D.

### 4.4 Post‐Congress Survey

Three days after the end of the congress, we advertised a post‐congress survey on the congress website and Twitter/X and e‐mailed this to people that registered for the congress or signed up for the newsletter. The survey was filled in by 391 people (approx. 45% of all attendees) and included sections with questions on (a) social identity (gender, pronouns, age, career stage, LGBTQ+, nationality, affiliation); (b) congress‐related questions on attendance; (c) self‐assessment of one's expertise and comfort speaking English; (d) conference experience; (e) question‐asking; (f) attendance of and feedback on EDI‐related activities such as the symposium and workshops; (g) perceived equality at the congress and in the field of behaviour, ecology and evolution in general; (h) childcare (was childcare used and how important was the offer for free childcare to the attendee); (i) disability (do you have a disability and was this adequately accommodated for) and (j) qualitative feedback. People that did not attend the congress were also able to fill in a shortened version of the survey that only asked for their social identity variables and reasons why they did not attend. As very few non‐attendees filled out the survey (*n* = 3), we do not report these results. At the start of the survey, respondents were asked to consent to their data being used for research, and answering the questions was optional.

Prior to the statistical analyses, we simplified and processed a number of variables obtained from the personal details section of the post‐congress survey (section a). First, we condensed the career stages into three categories: early‐career (BSc students, MSc students, postgraduates, and PhD students), mid‐career (finished PhD students awaiting a postdoctoral position, postdocs, lecturers, and researchers), late‐career (associate professors, assistant professors, and full professors) and “other” (applied scientists, non‐academics, retired scientists, technicians, etc.). We acknowledge that the categorisation of different positions into three career stages is not straight‐forward as the definition of a research position can vary across countries, and we would recommend future research to also collect information on people's academic age (e.g., time since their doctoral degree). Second, we added a variable expatriate status (“expat”), which indicated whether the country of affiliation is the same as the country of nationality (same = no expat, not the same = expat). We acknowledge that this variable is imperfect and only provides a contemporary snapshot of someone's expat status, yet it serves as an indicator of cultural exposure. Third, we categorised all countries (nationalities and affiliations) into the continents for simplification purposes and due to unequal and sometimes small sample sizes per country. People who indicated multiple countries of nationality (*n* = 6) were excluded from all analyses, as the countries were often located on different continents. From here onwards, we collectively refer to gender, career stage, sexual and gender identity (LGBTQ+), nationality, affiliation and expat status as the “social identity variables”. We also tested for the effect of expertise rating (Likert‐scale response to “I am an expert in my field”) and the effect of English comfort (Likert‐scale response to “I feel comfortable speaking in English”) and collectively refer to these variables as the “controlling” variables. Both of these responses were measured on a 7‐point Likert scale which indicated to what extent people agreed, ranging from “Strongly disagree” (1) to “Strongly agree” (7), where 4 would indicate a neutral attitude. Expertise rating might capture variation in non‐linear career pathways that career stage cannot account for (e.g., having worked in a corporate setting before pursuing an academic career). For clarity, a summary of the models that use the data collected in the post‐congress survey can be found in Table S15, which includes the research question it addresses and the formula written in lme4 syntax (Bates et al. 2015).

#### 4.4.1 Gender Effects on Question‐Asking Motivation and Hesitation

In section (e) of the post‐congress survey, we collected data on question‐asking behaviour. First, we asked whether participants asked one or multiple questions during the Q&A sessions at the congress (yes/no). We tested whether gender was predictive of a person having asked a question during the congress by fitting a binomial GLM to the response to this question as the dependent variable and using self‐reported gender as the independent variable. We used an LRT to evaluate whether gender was a significant predictor of the probability that a person asked a question.

Second, we asked which factor(s) motivated attendees to ask a question:

1. “Interest in the topic”
2. “Making my voice heard”
3. “Appraising the speaker's work”
4. “Deeper understanding”
5. “Showing the audience and speaker my understanding of the topic”
6. “Relevance for my own research”.

Next, we asked which factor(s) contributed to their hesitation to ask a question during the Q&A sessions:

1. “Not feeling clever enough”
2. “Afraid I misunderstood the content of the presentation”
3. “I felt intimidated by the speaker”
4. “I felt intimidated by the audience”
5. “I felt intimidated by the setting (e.g., size of the room)”
6. “I felt intimidated by the session chair”
7. “I did not think my question was relevant/important”
8. “Afraid I would not be able to phrase/articulate my question well”
9. “I did raise my hand but was not chosen to ask a question”
10. “There was no time left to ask my question”
11. “I am too much of an introvert”
12. “I would rather ask my question after the session one‐to‐one with the speaker”
13. “I did not have the confidence”

Note that hesitation number 9 is presented separately from the other hesitations in the results, as the response to this hesitation was used in combination with the observational data to understand whether women ask fewer questions because they were chosen less often by the session hosts than men.

Lastly, we presented a series of statements to identify which conditions might make people feel more comfortable to ask a question:

1. “I feel comfortable asking questions during Q&A sessions”
2. “I feel more comfortable asking questions to a speaker who is of my own gender”
3. “I feel more comfortable asking question when my own gender is represented in the audience”
4. “I feel more comfortable asking questions when the audience is smaller”
5. “I feel more comfortable asking questions when the session host is of my own gender”
6. “I feel more comfortable asking questions when I know the speaker”.

Similar to above, survey participants indicated on a 7‐point Likert scale to what extent they agreed with the six statements, where the scale ranged from “Strongly disagree” (1) to “Strongly agree” (7), where 4 would indicate a neutral attitude.

We built two sets of models to identify what motivations, hesitations, and conditions were more important for some gender identities than for others, and consequently which motivations, hesitations, and conditions were the best predictors of whether a person asked a question at the congress or not. First, we only selected motivations and hesitations that were ticked at least 15 times in general. Next, we identified which factors out of the selected motivations, hesitations, and conditions were significantly affected by gender. Separately for each motivation and hesitation, we then built binomial GLMs (PCS.1, Table S15) using the lme4 R package v1.1.35.3 (Bates et al. 2015). The binary response of whether this motivation or hesitation was applicable or not (1 = yes, 0 = no) was used as the dependent variable, and gender was used as an independent variable (female, male or non‐binary) as well as career stage (early‐, mid‐ or late‐career stage). For the ordinal condition responses, we built one ordered logistic regression (OLR) model for each one of the conditions with the R package MASS (Venables and Ripley 2002). To investigate whether gender had a significant effect on the response variable, we compared the fit of the model to a null model that only included the intercept using an LRT. We applied a multiple‐testing correction to all motivation, hesitation, and condition LRTs collectively using the false discovery rate (FDR, Benjamini and Hochberg 1995).

Next, we asked which of the motivations, hesitations, and conditions affected the probability that the person asked a question during the congress. For this, we built 24 separate binomial linear models (PCS.2, Table S15) using lme4, where the binary response, whether the person asked a question during the congress (1 = one or multiple questions asked, 0 = no questions asked), was used as a dependent variable and the response of the motivation/hesitation/condition as the independent variable. We further included both gender and career stage in the models to account for potential direct effects of these variables on the probability that a person asked a question independent from the motivation/hesitation/condition. Again, we evaluated whether the motivation/hesitation/condition had a significant effect on question‐asking probability using an LRT, which compared the fit of the model to a null model that only included the intercept and applied an FDR correction to the LRT outputs of all 24 models collectively.

#### 4.4.2 How Did Different Social Identities and People With Different Levels of Expertise and English Comfortability Experience the Conference?

We next identified which social identity and/or controlling variable(s) explained variation in congress experience. Post‐congress survey participants indicated on a 7‐point Likert scale (similar to above) to what extent they agreed with the following three statements about their congress experience:

1. “I felt heard during the conversations I had, both during Q&A sessions and social activities”
2. “I felt comfortable being myself”
3. “Attending the Behaviour 2023 congress helped me feel like I belong in my research field”

We built ordinal logistic regression (OLR) models of the responses to each of the three statements (PCS.3, PCS.4 and PCS.5, respectively, Table S15) using the *polr* function from the R package MASS (Venables and Ripley 2002). First, we identified which of the social identity variables significantly improved the fit of the models by fitting six separate models for each statement, with one of the social identity variables included as an independent variable. A significant social identity was identified using an LRT which compared the model that included the social identity variable to a null model that only included the intercept. In addition to identifying significant social identity variables, we also fitted expertise rating and English comfort rating as potential confounding variables and assessed if they improved the fit of the models using an LRT. Only variables that significantly improved the fit of the model (i.e., the *p‐value* of the LRT was less than 0.05) were included in the final model for that conference experience statement. We conducted a Wald test using the *coeftest* function from the R package lmtest v0.9‐40 (Zeileis and Hothorn 2002) to generate coefficients, standard errors and *p*‐values, and the *confint* function from the same package to generate the corresponding confidence intervals.

#### 4.4.3 Perceptions of Equity, Diversity and Inclusivity Among Congress Attendees (Statistical Analyses)

Similar to the analysis of congress experience, we investigated which social identity and/or controlling variable(s) explained variation in how attendees perceived EDI issues in the context of the congress and the broader research field. Survey participants indicated on a 7‐point Likert scale to what extent they agreed with three statements about perceived EDI issues:

1. “I think the Congress attendees represented the diversity of researchers in our field”
2. “Our research field experiences equity, diversity and inclusion‐related issues (e.g., racism, homophobia, harassment, bullying etc.)”
3. “I think the questions asked after the talks were equally divided across genders”

We took a similar approach as described above: (i) we fitted OLR models to the responses of each of the three EDI issue perception statements (PCS.6, PCS.7 and PCS.8, respectively, Table S15): (ii) we identified which of the social identity variable(s) were significantly associated with the response to the statement by conducting LRTs that compared the model for that social identity or controlling variable against a null model that did not include the variable; (iii) we built the final model to include only social identity variables that significantly improved the fit of the model. In addition to identifying significant social identity variables, we also fitted age and English comfort rating as potential confounding variables and assessed if they improved the fit of the models using an LRT.

#### 4.4.4 Qualitative Analysis of Open‐Ended Questions

In the post‐congress survey, participants were asked to respond to an open‐ended question with their feedback or opinions on the congress. Of the 391 total respondents, 48% (*n* = 191) provided a response to this question, of which 185 could be coded into their respective sentiments.

We used qualitative content analysis methodology (Schreier 2014) to code the open‐ended responses. Codes were assigned to the main elements (distinct pieces of information that convey a particular idea; e.g., organisation, provision for accessibility, etc.) in the responses. These elements were further tagged with the sentiments expressed as being ‘Positive’ (e.g., *well* organised, *good* focus on EDI), ‘Negative’ (e.g., *tight* schedule/*inadequate* scheduling, *inadequate* provisions for accessibility) or providing a ‘Suggestion’ (e.g., *alternative* scheduling, search function in abstracts). Since multiple respondents provided extensive responses to the question, each response could therefore have more than one code and/or sentiment expressed in it, leading to double counts. This preliminary coding was done by two independent people (both members of the research team) who coded all of the responses. The coders then discussed misalignments in coding until a consensus was achieved for all of the responses. At the end of this phase, we had 824 coded elements across 78 codes. These codes were then aggregated based on their similarity. At the end of this phase, we had 24 codes (8 in each sentiment).

All statistical analyses were implemented in R v.4.3.2 (Team 2021) using RStudio v. 2023.09.1. Data were visualised using the packages ggplot2 v3.5.1 (Wickham 2016), cowplot v1.1.3 (Wilke 2024) and viridis v0.6.5 (Garnier et al. 2024).

## Author Contributions

**Rebecca S. Chen:** conceptualization (equal), data curation (equal), formal analysis (equal), funding acquisition (equal), investigation (equal), methodology (lead), project administration (equal), supervision (lead), visualization (equal), writing – original draft (lead), writing – review and editing (equal). **Tuba Rizvi:** conceptualization (equal), formal analysis (equal), funding acquisition (equal), investigation (equal), methodology (equal), visualization (equal), writing – original draft (supporting), writing – review and editing (equal). **Ane Liv Berthelsen:** formal analysis (equal), investigation (equal), visualization (equal), writing – review and editing (equal). **Anneke J. Paijmans:** investigation (equal), methodology (equal), writing – review and editing (equal). **Avery L. Maune:** formal analysis (equal), investigation (equal), visualization (equal), writing – review and editing (equal). **Barbara A. Caspers:** methodology (equal), writing – review and editing (equal). **Bernice Sepers:** formal analysis (equal), investigation (equal), methodology (equal), visualization (equal), writing – original draft (supporting), writing – review and editing (equal). **Isabel Damas‐Moreira:** investigation (equal), writing – review and editing (equal). **Isabel Schnülle:** writing – review and editing (equal). **Jana Könker:** investigation (equal), methodology (equal), writing – review and editing (equal). **Joseph I. Hoffman:** investigation (equal), methodology (equal), writing – review and editing (equal). **Joelyn de Lima:** formal analysis (equal), methodology (equal), visualization (equal), writing – review and editing (equal). **Jonas Tebbe:** formal analysis (equal), investigation (equal), methodology (equal), visualization (equal), writing – review and editing (equal). **Kai‐Philipp Gladow:** investigation (equal), methodology (equal), writing – review and editing (equal). **Lisa de Vries:** writing – review and editing (equal). **Marc Gilles:** formal analysis (equal), investigation (equal), methodology (equal), visualization (equal), writing – review and editing (equal). **Nadine Schubert:** project administration (equal), writing – review and editing (equal). **Nayden Chakarov:** investigation (equal), methodology (equal), writing – review and editing (equal). **Peter Korsten:** investigation (equal), methodology (equal), writing – review and editing (equal). **Petroula Botsidou:** formal analysis (equal), investigation (equal), methodology (equal), visualization (equal), writing – review and editing (equal). **Sabine Kraus:** formal analysis (equal), investigation (equal), methodology (equal), visualization (equal), writing – review and editing (equal). **Stephen M. Salazar:** investigation (equal), methodology (equal), writing – review and editing (equal). **Svenja Stöhr:** investigation (equal), writing – review and editing (equal). **Wolfgang Jockusch:** writing – review and editing (equal). **Öncü Maraci:** conceptualization (equal), funding acquisition (equal), investigation (equal), writing – review and editing (equal).

## Ethics Statement

This study has been approved by the Ethics Committee of Bielefeld University under application number 2023‐140. Informed consent was obtained for both the pre‐ and post‐congress surveys.

## Conflicts of Interest

The authors declare no conflicts of interest.

## Supporting information


Table S1.–S15.

## Figures and Tables

**FIGURE A1:**
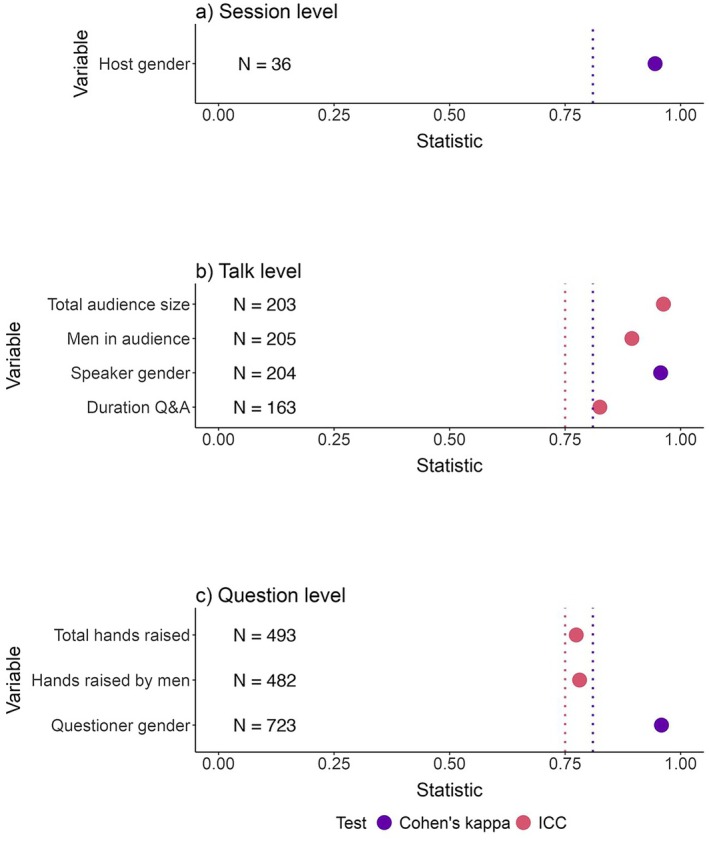
Inter‐observer reliability statistics for each variable collected on the three different levels. Cohen's Kappa and ICC statistics calculated for variables collected per session (a), talk (b) and question (c). Vertical purple and red dotted lines indicate commonly accepted thresholds for Cohen's kappa (Cohen's kappa > 0.8 = “near perfect”; Cohen 1968) and ICC (ICC > 0.75 = “good”; Koo and Li 2016) respectively.

## Data Availability

All anonymised data for the pre‐congress survey, question‐asking behaviour and post‐congress survey can be found on https://github.com/rshuhuachen/ms_edi_behaviour23 and Zenodo https://zenodo.org/records/13825175 with DOI 10.5281/zenodo.13825175. These repositories also include all code used to analyse the data and additional documents shared to increase transparency and reproducibility, such as the Code of Conduct and the protocol used for collecting data on question‐asking behaviour. Although all respondents of the post‐congress survey consented to their data being used for research anonymously, we did not publish the qualitative feedback that was part of the survey, as anonymity cannot be guaranteed. A summary of the entire workflow, including the code and results, can be found on https://rshuhuachen.github.io/ms_edi_behaviour23/.

## Appendix A Manual Curation of Behavioural Data

Data collection sheets were digitised by a team of nine student assistants. Despite the training of observers, complex situations occurred that were not anticipated, or situations were interpreted differently by the observers that sampled the same session. Consequently, inconsistencies between two observers sometimes occurred, for example in records of the number of questions that were asked during a Q&A. Because inconsistencies in the number of questions asked in a session made it difficult if not impossible to match up the data collected by two observers in the same session, we manually resolved these inconsistencies based on the notes taken. To ensure this manual curation was reliable and did not introduce mistakes based on subjective interpretations by single people, two data curators assessed the inconsistencies independently. The most common reason for disagreements in the number of questions asked was due to one questioner asking multiple questions, which was noted down inconsistently by observers. We manually corrected for this by adding a note on whether a question was a follow‐up question (defined as a question that was asked by the same person consecutively, i.e., without a question being asked by another person in between) and excluded these follow‐up questions in our analyses.

In addition, there were certain sessions where collecting data was more difficult, for example when the room was very busy, making it difficult to estimate the audience size, or when the lighting in the room was suboptimal, making it difficult to estimate a person's age or infer their gender. We added an additional binomial parameter to each datapoint to indicate whether there was any kind of uncertainty in the data collected, based on the notes taken during that session/talk/question. This allowed us to implement a conservative analytical approach in which we compared the results of models that included and excluded these ‘unreliable’ data that included potential biases. The dataset excluding unreliable data of any kind is hereafter referred to as the “conservative dataset”.

## Appendix B Inter‐Observer Reliability

In 32 out of 67 sessions, multiple observers collected data on question‐asking behaviour to quantify the reliability of our observations and consequently, the credibility of our data. To evaluate inter‐observer reliability (IOR), we calculated the unweighted Cohen's kappa (Cohen 1968) for nominal variables and the intraclass correlation coefficient (ICC) for numeric variables using a two‐way agreement model implemented in the R package irr v.0.84.1 (Gamer et al. 2005). The sessions that were double‐sampled and took place in large lecture rooms were sampled by four observers, with the role of counting the number of men and people in total that raised their hands to ask a question being split between a pair of two observers. Thus, in double‐sampled sessions in large lecture rooms, all of the variables other than the number of hands raised were recorded by two pairs of observers rather than a single pair. Because the IOR statistic is calculated for a given number of observers and we aimed to calculate this statistic across all sessions regardless of room size, we treated the two pairs in large lecture rooms as two independent double‐samples and thus only tested for the agreement within pairs and not between pairs.

A Cohen's kappa value between 0.40 and 0.60 is interpreted as “moderate” agreement, a value between 0.61 and 0.80 is interpreted as “substantial” agreement, and a value over 0.80 is interpreted as “near perfect” (Cohen 1968). An ICC between 0.50 and 0.75 indicates “moderate” reliability, whereas a value between 0.75 and 0.90 indicates “good” reliability, and above 0.90 “excellent” (Koo and Li 2016). Observers had an “almost perfect” agreement on gender (host gender Cohen's kappa = 0.94, *p* < 0.001; speaker gender Cohen's kappa = 0.96, *p* < 0.001; questioner gender Cohen's kappa = 0.96, *p* < 0.001) and audience size (total audience size ICC = 0.96, *p* < 0.001; men in audience ICC 0.89, *p* < 0.001) and a “good” agreement on the duration of the Q&A (ICC = 0.83, *p* < 0.001). There was a “good” agreement on the number of hands raised in total (ICC = 0.77, *p* < 0.001) and by men only (ICC = 0.78, *p* < 0.001). The results are visualised in Figure A1.

## Appendix C Merging Data From Double‐Sampled Sessions

We combined the different observations of each parameter recorded in double‐sampled sessions based on the conditions noted down in Table A1 below. Due to the importance of gender for our analyses and the sensitivity of these data, we excluded any data points where the observers disagreed on this variable. If there was inconsistency on the noted number of hands raised by different observers, the most plausible explanation is that one of the two observers did not see one of the hands raised, and therefore we took the maximum number of raised hands. Similarly, if one observer noted down that a compliment was given to the speaker or that the speaker talked for longer than instructed, and the other observer did not, the most likely cause is that the other observer did not notice this or forgot to note it down. Lastly, interpreting a question as a challenge to the speaker might depend on the observer's expertise on the subject and/or conscious and unconscious bias in interpreting the questioner, which might lead to two observers interpreting the question differently. However, if one of the two observers interpreted the question as challenging, it is likely that at least part of the audience as well as the speaker also ‘felt’ this. Therefore, we applied the same logic as above and only one of the two observers had to note the question down as challenging for us to include this in the curated dataset.

**TABLE A1:** Conditional workflow used to combine data collected by different observers of the same session.

Variable	How data were combined
Gender questioner/speaker/host	If observers disagree, set to N/A
Audience size	Mean of the audience sizes, if the disagreement was high (SD > 20), put both audience sizes to N/A
Duration Q&A session	Mean of the durations
Number of hands raised	The maximum number of hands raised
Was a compliment given?	If one of the observers said yes, then yes
Did the speaker talk for longer than the allocated time slot?	If one of the observers said yes, then yes
Was the question type ‘challenging’?	If one of the observers said yes, then yes

## Appendix D Other Gender Disparities in Q&A Sessions

In addition to identifying a gender disparity in asking questions, we asked if there were gender disparities in other aspects of the oral sessions that were related to the content of the question, waiting for your turn to ask a question, and accurately timing your talk. First, we found that neither the perceived gender of the speaker (LRT *p* = 0.14) nor the perceived gender of the questioner (LRT *p* = 0.23) significantly affected the probability that a compliment was given. The probability that a person gave a compliment was, however, highest at the start of the Q&A (estimated question number = −0.34, *p* < 0.001). The perceived gender of the speaker (LRT *p* = 0.25) nor the perceived gender of the questioner (LRT *p* = 0.35) affected the probability of receiving a critical question. Next, we found that jumping a question (i.e., asking a question without being chosen to do so) did not occur frequently (*n* = 18), and there was no significant effect of perceived questioner gender on the probability that the person jumped a question (LRT *p* = 0.10). Also, the perceived gender of the host did not affect the probability of a jumper (LRT *p* = 0.57). Lastly, the perceived gender of the speaker did not affect the probability that the speaker talked for longer than their allocated time slot (LRT *p* = 0.78).
