# Supplementary material for: How Can We Make Scientific Events More Inclusive? Insights From Q&A Sessions and Surveys From an International Conference
Source: Ecol Evol. 2025 Jul 13;15(7):e71588. doi: 10.1002/ece3.71588 (PMC12256129; doi:10.1002/ece3.71588)
Supplement: Supplementary file 1 — Table S1.–S15. [file ECE3-15-e71588-s001.docx]

**Supporting Information**

**Supporting Tables**

**Table S1. Model output for question-asking models based on both behavioural and survey data.** The models investigated gender disparities in question-asking (QA), raising hands (RH) or being chosen to ask a question (GC), where some QA models were given a name in the Methods and are indicated in the table in parenthesis. For the models using behavioural data (BD), the intercepts are indicative of a gender disparity. For the models using survey data (SD), we tested whether including gender improved model fit using an LRT and additionally computed a Wald test. Bold numbers indicate statistical significance (*p* < 0.05). One observation in the BD represents a single question, whereas one observation in the SD represents a single response. Abbreviations: O = observations, T = talks, IC = intercept.

| **Model** | **Data** | **# O** | **LRT χ^2^** | **LRT *p*** | **Reference level** | **Term** | **Estimate ± SE** | ***z*** | **Wald test *p*** |
| --- | --- | --- | --- | --- | --- | --- | --- | --- | --- |
| QA (QA.1) | BD | 350 (127 T) | N/A | N/A | N/A | IC | -0.66 ± 0.11 | -6.07 | < 0.001 |
| QA **(QA.1c)** | BD – conser-vative | 60 (124 T) | N/A | N/A | N/A | IC | -0.67 ± 0.11 | -6.12 | < 0.001 |
| QA (QA.1o) | BD – only double-sampled sessions | 119 (46 T) | N/A | N/A | N/A | IC | -0.43 ± 0.19 | -2.32 | 0.02 |
| QA **(QA.1p)** | BD – plenary | 342 (10 T) | N/A | N/A | N/A | IC | -1.54 ± 0.31 | -4.95 | < 0.001 |
| QA | SD | 373 | 5.96 | 0.05 | Male | Woman | -0.49 ± 0.24 | -2.01 | 0.04 |
|  |  |  |  |  |  | Non-binary | 0.92 ± 1.10 | 0.84 | 0.40 |
| RH (QA.2) | BD | 349 (127 T) | N/A | N/A | N/A | IC | -0.58 ± 0.11 | -5.45 | < 0.001 |
| GC (QA.3) | BD | 99 (67 T) | N/A | N/A | N/A | IC | -0.14 ± 0.23 | -0.62 | 0.53 |
| GC | SD | 375 | 1.49 | 0.48 | Male | Woman | 0.26 ± 0.33 | 0.78 | 0.44 |
|  |  |  |  |  |  | Non-binary | 1.03 ± 0.88 | 1.17 | 0.24 |

**Table S2. Model output for gender differences in question-asking motivation and probability based on post-congress survey data.** We first tested which motivations were significantly influenced by gender using LRTs and consequently conducted Wald tests (left). We next tested which motivations were predictive of the probability that a person asked a question during the congress, also using LRTs and Wald tests (right). Bold numbers indicate statistical significance (*p* < 0.05).

| **Motivation** | **Gender effect on motivation** | | | | | **Motivation effect on probability of asking a question** | | | | |
| --- | --- | --- | --- | --- | --- | --- | --- | --- | --- | --- |
|  | LRT χ^2^ | LRT FDR-*q* | Woman estimate ± SE | *z* | Wald test *p* | LRT χ^2^ | LRT FDR-*q* | Estimate ± SE | *z* | Wald test *p* |
| Relevance own research | 1.29 | 0.61 | 0.07 ± 0.23 | 0.31 | 0.75 | 0.03 | 0.91 | -0.04 ± 0.23 | -0.17 | 0.87 |
| Making voice heard | 5.95 | 0.13 | -0.60 ± 0.46 | -1.30 | 0.19 | 7.03 | 0.02 | 1.68 ± 0.76 | 2.20 | 0.02 |
| Interest in topic | 4.05 | 0.23 | -0.53 ± 0.38 | -1.39 | 0.17 | 10.32 | 0.00 | 1.07 ± 0.34 | 3.15 | 0.00 |
| Deeper under-standing | 0.84 | 0.69 | -0.05 ± 0.26 | -0.19 | 0.85 | 3.94 | 0.09 | 0.51 ± 0.26 | 1.98 | 0.08 |
| Appreciate work | 0.98 | 0.68 | -0.24 ± 0.27 | -0.91 | 0.37 | 2.25 | 0.23 | 0.43 ± 0.29 | 1.48 | 0.19 |

**Table S3.** **Model output for gender differences in question-asking hesitation and probability based on post-congress survey data.** We first tested which hesitations were significantly influenced by gender using LRTs and consequently conducted Wald tests (left). We next tested which hesitations were predictive of the probability that a person asked a question during the congress, also using LRTs and Wald tests (right). Bold numbers indicate statistical significance (*p* < 0.05).

| **Hesitation** | **Gender effect on hesitation** | | | | | **Hesitation effect on probability of asking a question** | | | | |
| --- | --- | --- | --- | --- | --- | --- | --- | --- | --- | --- |
|  | LRT χ^2^ | LRT FDR-*q* | Woman estimate ± SE | *z* | Wald test *p* | LRT χ^2^ | LRT FDR-*q* | Estimate ± SE | *z* | Wald test *p* |
| Too introverted | 4.04 | 0.23 | 0.52 ± 0.29 | 1.80 | 0.07 | 23.12 | 0.00 | -1.27 ± 0.27 | -4.72 | < 0.001 |
| Rather in private | 5.37 | 0.16 | 0.30 ± 0.23 | 1.28 | 0.20 | 19.84 | 0.00 | -1.04 ± 0.24 | -4.38 | < 0.001 |
| Phrasing | 11.19 | 0.02 | 0.90 ± 0.29 | 3.16 | 0.00 | 1.80 | 0.24 | -0.34 ± 0.25 | -1.35 | 0.18 |
| Not clever | 2.74 | 0.33 | 0.42 ± 0.26 | 1.61 | 0.11 | 5.32 | 0.04 | -0.56 ± 0.24 | -2.31 | 0.02 |
| No time | 3.78 | 0.20 | -0.40 ± 0.25 | -1.61 | 0.11 | 12.41 | 0.00 | 0.98 ± 0.29 | 3.38 | < 0.001 |
| No con-  fidence | 7.64 | 0.08 | 0.78 ± 0.31 | 2.53 | 0.01 | 6.80 | 0.02 | -0.70 ± 0.27 | -2.64 | 0.01 |
| Mis-  understand | 0.36 | 0.83 | 0.04 ± 0.24 | 0.15 | 0.88 | 0.04 | 0.87 | -0.05 ± 0.24 | -0.21 | 0.84 |
| Irrelevance/un-  important | 3.89 | 0.23 | -0.26 ± 0.23 | -1.14 | 0.26 | 0.85 | 0.44 | 0.22 ± 0.23 | 0.92 | 0.36 |
| Intimida-  tion setting | 4.87 | 0.18 | 0.72 ± 0.47 | 1.53 | 0.13 | 0.18 | 0.76 | -0.17 ± 0.40 | -0.43 | 0.67 |
| Intimida-  tion audience | 6.27 | 0.13 | 0.76 ± 0.33 | 2.31 | 0.02 | 7.33 | 0.02 | -0.77 ± 0.28 | -2.70 | 0.01 |

**Table S4.** **Model output for age differences in question-asking motivation and hesitation based on post-congress survey data.** This output is based on the same models presented in Table S2 and Table S3, but here we report the estimates of the career stages. The reference level was the early-career stage, against which both mid- and late-career stages were compared. We only conducted a Wald test and did not correct for multiple testing, as career stage was not our main variable of interest (but gender was). Bold numbers indicate statistical significance (*p* < 0.05).

|  | **Motivation or hesitation** | **Career stage** | **Estimate ± SE** | ***z*** | **Wald test *p*** |
| --- | --- | --- | --- | --- | --- |
| Motivations | Relevance own research | Mid-career | 0.10 ± 0.23 | 0.42 | 0.67 |
|  |  | Late-career | -0.05 ± 0.33 | -0.16 | 0.88 |
|  | Making voice heard | Mid-career | 0.86 ± 0.51 | 1.67 | 0.09 |
|  |  | Late-career | 0.7 ± 0.73 | 0.97 | 0.33 |
|  | Interest in topic | Mid-career | -0.53 ± 0.33 | -1.61 | 0.11 |
|  |  | Late-career | 1.73 ± 1.04 | 1.67 | 0.10 |
|  | Deeper understanding | Mid-career | 0.21 ± 0.26 | 0.80 | 0.43 |
|  |  | Late-career | -0.45 ± 0.35 | -1.27 | 0.20 |
|  | Appreciate work | Mid-career | 0.14 ± 0.28 | 0.51 | 0.61 |
|  |  | Late-career | 0.5 ± 0.38 | 1.30 | 0.19 |
| Hesitations | Too introverted | Mid-career | -0.35 ± 0.27 | -1.30 | 0.19 |
|  |  | Late-career | -0.96 ± 0.47 | -2.04 | 0.04 |
|  | Rather in private | Mid-career | -0.05 ± 0.23 | -0.21 | 0.84 |
|  |  | Late-career | -1.11 ± 0.38 | -2.92 | < 0.001 |
|  | Phrasing | Mid-career | -0.57 ± 0.25 | -2.25 | 0.02 |
|  |  | Late-career | -1.59 ± 0.5 | -3.16 | < 0.001 |
|  | Not clever | Mid-career | -0.82 ± 0.25 | -3.26 | < 0.001 |
|  |  | Late-career | -1.79 ± 0.5 | -3.58 | < 0.001 |
|  | No time | Mid-career | 0.55 ± 0.26 | 2.12 | 0.03 |
|  |  | Late-career | 1.20 ± 0.35 | 3.41 | < 0.001 |
|  | No confidence | Mid-career | -0.42 ± 0.27 | -1.56 | 0.12 |
|  |  | Late-career | -2.91 ± 1.03 | -2.84 | 0.01 |
|  | Misunderstand | Mid-career | -0.96 ± 0.24 | -4.00 | < 0.001 |
|  |  | Late-career | -1.50 ± 0.4 | -3.72 | 0.00 |
|  | Irrelevance/un-important | Mid-career | -0.33 ± 0.23 | -1.40 | 0.16 |
|  |  | Late-career | -0.71 ± 0.36 | -1.96 | 0.05 |
|  | Intimidation setting | Mid-career | -0.97 ± 0.48 | -2.02 | 0.04 |
|  |  | Late-career | 0.29 ± 0.51 | 0.58 | 0.56 |
|  | Intimidation audience | Mid-career | -1.21 ± 0.32 | -3.76 | 0.00 |
|  |  | Late-career | -1.2 ± 0.51 | -2.37 | 0.02 |

**Table S5. Model output for variables affecting question-asking probability of perceived women using the behavioural data.** We tested whether including each variable significantly improves the model fit using a likelihood ratio test (LRT) and additionally report model output of the Wald test.

| **Variable** | **LRT χ^2^** | **LRT FDR-*q*** | **Estimate ± SE** | ***z*** | **Wald test *p*** |
| --- | --- | --- | --- | --- | --- |
| Speaker perceived as a woman (QA.1a) | 0.00 | 0.98 | -0.00 ± 0.20 | -0.02 | 0.98 |
| Proportion of audience that’s perceived as a woman (QA.1b) | 2.03 | 0.15 | -1.74 ± 1.20 | -1.44 | 0.15 |
| Host perceived as a woman (QA.1c) | 1.52 | 0.22 | 0.30 ± 0.24 | 1.21 | 0.23 |
| Audience size (QA.1d) | 0.06 | 0.81 | -0.00 ± 0.00 | -0.23 | 0.81 |
| Small room (compared to large room) (QA.1e) | 1.43 | 0.49 | -0.15 ± 0.23 | -0.65 | 0.51 |

**Table S6. Model output for gender effects on feeling comfortable asking questions using the post-congress survey data.** We tested whether including gender significantly improved model fits using LRTs and additionally report model output for the estimates of self-identified women compared to self-identified men gender using Wald tests. Bold numbers indicate statistical significance (*p <* 0.05).

| **Response (Likert-scale)** | **LRT χ^2^** | **LRT FDR-*q*** | **Level** | **Estimate ± SE** | ***t*** | **Wald test *p*** |
| --- | --- | --- | --- | --- | --- | --- |
| Audience is of own gender | 41.06 | < 0.001 | Women | 1.33 ± 0.22 | 6.05 | < 0.001 |
|  |  |  | Non-binary | 1.90 ± 0.66 | 2.88 | 0.004 |
| Speaker is of own gender | 36.30 | < 0.001 | Women | 1.22 ± 0.23 | 5.40 | < 0.001 |
|  |  |  | Non-binary | 2.44 ± 0.68 | 3.58 | < 0.001 |
| Host is of own gender | 19.64 | < 0.001 | Women | 0.92 ± 0.22 | 4.11 | < 0.001 |
|  |  |  | Non-binary | 1.58 ± 0.67 | 2.34 | 0.02 |
| Audience size is smaller | 15.81 | < 0.001 | Women | 0.79 ± 0.21 | 3.84 | < 0.001 |
|  |  |  | Non-binary | -0.07 ± 0.67 | -0.10 | 0.91 |

**Table S7. Model output testing gender effects of the first questioner on the probability that a woman asks a question in the rest of the Q&A using all questions (except the first one, Q1).** We first tested whether including the condition significantly improved the model fit using LRTs and additionally report model output of Wald tests. Bold numbers indicate statistical significance (*p* < 0.05). Abbreviations: T = talks, Q = questions, W = woman, M = man, P = probability.

| **Data** | **Model** | **#T, #Q** | **LRT χ^2^** | **LRT *p*** | **Condition** | **Estimate ± SE** | **P** | ***z*** | **Wald test *p*** |
| --- | --- | --- | --- | --- | --- | --- | --- | --- | --- |
| Unmani-pulated, all Q minus Q1 | Question- asking (QA.4**.**u) | 96, 212 | 6.34 | 0.01 | W first | -1.04 ± 0.19 | 0.26 | -5.38 | < 0.001 |
|  |  |  |  |  | M first | -0.33 ± 0.21 | 0.42 | -1.57 | 0.12 |
|  | Raising hands (QA.5**.**u) | 96, 209 | 4.90 | 0.03 | W first | -0.90 ± 0.20 | 0.29 | -4.62 | < 0.001 |
|  |  |  |  |  | M first | -0.31 ± 0.22 | 0.42 | -1.42 | 0.16 |
|  | Getting chosen (QA.6**.**u) | 37, 51 | 0.11 | 0.74 | W first | -0.13 ± 0.36 | 0.47 | -0.36 | 0.72 |
|  |  |  |  |  | M first | -0.33 ± 0.47 | 0.42 | -0.71 | 0.48 |
| Mani-  pulated, all Q minus Q1 | Question- asking (QA.4**.**m) | 90, 220 | 2.14 | 0.14 | W first | -0.66 ±  0.20 | 0.34 | -3.22 | 0.001 |
|  |  |  |  |  | M first | -0.25 ± 0.19 | 0.44 | -1.34 | 0.18 |
|  | Raising hands (QA.5**.**m) | 85, 204 | 1.32 | 0.25 | W first | -0.92 ± 0.20 | 0.29 | -4.51 | < 0.001 |
|  |  |  |  |  | M first | -0.62 ± 0.18 | 0.35 | -3.52 | < 0.001 |
|  | Getting chosen (QA.6**.**m) | 32, 49 | 0.01 | 0.91 | W first | 0.61 ± 0.45 | 0.65 | 1.37 | 0.17 |
|  |  |  |  |  | M first | 0.68 ± 0.42 | 0.66 | 1.63 | 0.10 |

**Table S8. Model output testing gender effects of the first questioner on the probability that a woman asks the second question (Q2).** We first tested whether including condition (first question to a woman or first question to a man) significantly improved the model fit using LRTs and additionally report model output of Wald tests. Bold numbers indicate statistical significance (*p* < 0.05). Abbreviations: T = talks, Q = questions, W = woman, M = man, P = probability.

| **Data** | **Model** | **# T, # Q** | **LRT χ^2^** | **LRT *p*** | **Condition** | **Estimate ± SE** | **P** | ***z*** | **Wald test *p*** |
| --- | --- | --- | --- | --- | --- | --- | --- | --- | --- |
| Unmani-pulated, Q2 | Question- asking (QA.4.u.2) | 76, 76 | 5.68 | 0.02 | W first | -1.30 ± 0.36 | 0.21 | -3.59 | < 0.001 |
|  |  |  |  |  | M first | -0.15 ± 0.35 | 0.46 | -0.43 | 0.67 |
|  | Raising hands (QA.5.u.2) | 75, 75 | 7.01 | 0.008 | W first | -1.08 ± 0.23 | 0.25 | -4.78 | < 0.001 |
|  |  |  |  |  | M first | -0.14 ± 0.28 | 0.46 | -0.52 | 0.60 |
|  | Getting chosen (QA.6.u.2) | 26, 26 | 0.02 | 0.90 | W first | -0.25 ± 0.56 | 0.44 | -0.45 | 0.65 |
|  |  |  |  |  | M first | -0.36 ± 0.66 | 0.41 | -0.55 | 0.58 |
| Mani-  pulated, Q2 | Question -asking (QA.4.m.2) | 75, 75  Model failed to converge | N/A | N/A | W first | N/A | N/A | N/A | N/A |
|  |  |  |  |  | M first | N/A | N/A | N/A | N/A |
|  | Raising hands (QA.5.m.2) | 71, 71 | 2.21 | 0.14 | W first | -0.93 ± 0.31 | 0.28 | -3.00 | 0.003 |
|  |  |  |  |  | M first | -0.32 ± 0.29 | 0.42 | -1.08 | 0.28 |
|  | Getting chosen (QA.6.m.2) | 20, 20 | 2.33 | 0.13 | W first | 0.04 ± 0.69 | 0.51 | 0.05 | 0.96 |
|  |  |  |  |  | M first | 1.62 ± 1.05 | 0.84 | 1.54 | 0.12 |

**Table S9. Model output for the three statements on congress experience**. Univariate models tested for the significance of each variable using LRTs and only variables that significantly improved the model fit (indicated in bold) were included in the final model. Bold numbers indicate statistical significance (*p* < 0.05).

|  | **Variable** | **Univariate models** | | **Final models** | | | | | |
| --- | --- | --- | --- | --- | --- | --- | --- | --- | --- |
|  |  | **LRT χ^2^** | **LRT *p*** | **Level** | **Estimate ± SE** | | ***t*** | | **Wald test *p*** |
| **Feeling heard (PCS.3)** | Gender | 4.38 | 0.11 | N/A | | | | | |
|  | LGBTQ+ | 3.57 | 0.06 |  |  |  |  |  |  |
|  | Nationality | 9.24 | 0.06 |  |  |  |  |  |  |
|  | Affiliation | 8.70 | 0.12 |  |  |  |  |  |  |
|  | Expat | 1.66 | 0.20 |  |  |  |  |  |  |
|  | English comfort | 14.38 | < 0.001 | N/A | | 0.28 ± 0.10 | 2.77 | 0.006 | |
|  | Expertise | 21.91 | < 0.001 | N/A | | 0.24 ± 0.06 | 3.85 | < 0.001 | |
| **Comfortable being myself (PCS.4)** | Gender (relative to men) | 13.30 | 0.001 | Woman | | -0.48 ± 0.22 | -2.14 | 0.03 | |
|  |  |  |  | Non-binary | | -2.26 ± 0.68 | -3.35 | 0.001 | |
|  | LGBTQ+ | 3.30 | 0.07 | N/A | | | | | |
|  | Nationality | 2.01 | 0.74 |  |  |  |  |  |  |
|  | Affiliation | 6.17 | 0.29 |  |  |  |  |  |  |
|  | Expat | 0.01 | 0.95 |  |  |  |  |  |  |
|  | English comfort | 10.60 | 0.001 | N/A | | 0.28 ± 0.11 | 2.56 | 0.01 | |
|  | Expertise | 17.77 | < 0.001 | N/A | | 0.22 ± 0.06 | 3.53 | < 0.001 | |
| **Sense of belonging (PCS.5)** | Gender | 4.48 | 0.11 | N/A | | | | | |
|  | LGBTQ+ | 0.82 | 0.37 |  |  |  |  |  |  |
|  | Nationality | 7.50 | 0.11 |  |  |  |  |  |  |
|  | Affiliation  (relative to  Europe) | 14.46 | 0.01 | Asia | | 0.88 ± 0.52 | 1.71 | 0.09 | |
|  |  |  |  | Africa | | -1.02 ± 1.48 | -0.69 | 0.49 | |
|  |  |  |  | North America | | 1.16 ± 0.53 | 2.19 | 0.03 | |
|  |  |  |  | Oceania | | 0.06 ± 0.52 | 0.12 | 0.90 | |
|  |  |  |  | South America | | 15.76 ± 0.0 | Inf | < 0.001 | |
|  | Expat | 0.52 | 0.47 | N/A | | | | | |
|  | English comfort | 19.43 | < 0.001 | N/A | | 0.31 ± 0.10 | 3.03 | 0.003 | |
|  | Expertise | 45.30 | < 0.001 | N/A | | 0.35 ± 0.06 | 0.06 | < 0.001 | |

**Table S10. Model output for the three statements on EDI issue perception**. Univariate models tested for the significance of each variable using LRTs and only variables that significantly improved the model fit (indicated in bold) were included in the final model. Bold numbers indicate statistical significance (*p* < 0.05).

|  | **Variable** | **Univariate models** | | **Final models** | | | |
| --- | --- | --- | --- | --- | --- | --- | --- |
|  |  | **LRT χ^2^** | **LRT *p*** | **Level** | **Estimate ± SE** | ***t*** | **Wald test *p*** |
| **Attendee diversity (PCS.6)** | Gender (relative to male) | 9.05 | 0.01 | Woman | -0.53 ± 0.21 | -2.56 | 0.01 |
|  |  |  |  | Non-binary | -0.83 ± 0.68 | -1.22 | 0.22 |
|  | LGBTQ+ | 6.95 | 0.01 | LGBTQ+ | -0.60 ± 0.28 | -2.18 | 0.03 |
|  | Nationality | 4.02 | 0.40 | N/A | | | |
|  | Affiliation | 3.96 | 0.55 |  |  |  |  |
|  | Expat | 1.31 | 0.25 |  |  |  |  |
|  | English comfort | 0.77 | 0.38 |  |  |  |  |
|  | Age | 1.23 | 0.54 |  |  |  |  |
| **EDI issues (PCS.7)** | Gender (relative to male) | 10.92 | < 0.01 | Woman | 0.48 ± 0.22 | 2.20 | 0.03 |
|  |  |  |  | Non-binary | 0.24 ± 0.69 | 0.34 | 0.73 |
|  | LGBTQ+ | 10.40 | 0.001 | LGBTQ+ | 0.73 ± 0.28 | 2.64 | < 0.01 |
|  | Nationality  (relative to Europe) | 12.39 | 0.02 | Asia | -0.34 ± 0.34 | -0.98 | 0.33 |
|  |  |  |  | North America | 0.77 ± 0.35 | 2.22 | 0.03 |
|  |  |  |  | Oceania | 0.37 ± 0.69 | 0.54 | 0.59 |
|  |  |  |  | South America | 1.27 ± 0.80 | 1.59 | 0.11 |
|  | Affiliation | 6.78 | 0.24 | N/A | | | |
|  | Expat | 8.88 | < 0.01 | Expat | 0.55 ± 0.20 | 2.76 | 0.01 |
|  | English comfort | 0.30 | 0.58 | N/A | | | |
|  | Age | 2.52 | 0.28 |  |  |  |  |
| **No QA gender disparity (PCS.8)** | Gender (relative to male) | 8.58 | 0.01 | Woman | -0.41 ± 0.22 | -1.81 | 0.07 |
|  |  |  |  | Non-binary | -1.08 ± 0.70 | -1.55 | 0.12 |
|  | LGBTQ+ | 7.60 | < 0.01 | LGBTQ+ | -0.52 ± 0.29 | -1.80 | 0.07 |
|  | Nationality (relative to Europe) | 13.09 | 0.01 | Asia | 0.74 ± 0.45 | 1.64 | 0.10 |
|  |  |  |  | North America | 0.43 ± 0.42 | 1.03 | 0.30 |
|  |  |  |  | Oceania | -0.26 ± 0.87 | -0.30 | 0.77 |
|  |  |  |  | South America | 2.64 ± 1.30 | 2.04 | 0.04 |
|  | Affiliation  (relative to  Europe) | 15.32 | < 0.01 | Asia | 0.58 ± 0.70 | 0.83 | 0.41 |
|  |  |  |  | Africa | 1.74 ± 1.51 | 1.16 | 0.25 |
|  |  |  |  | North America | -0.45 ± 0.50 | -0.91 | 0.37 |
|  |  |  |  | Oceania | 0.53 ± 0.78 | 0.68 | 0.50 |
|  |  |  |  | South America | -5.39 ± 1.95 | -2.76 | 0.01 |
|  | Expat | 0.06 | 0.80 | N/A | | | |
|  | English comfort | 5.80 | 0.01 | N/A | -0.23 ± 0.11 | -2.23 | 0.03 |
|  | Age | 3.51 | 0.17 | N/A | | | |

| **Table S11. Codes used for the qualitative analysis of open text responses.** Both condensed and expended codes are presented as well as their frequency the codes were expressed in the responses. | | | | |
| --- | --- | --- | --- | --- |
| **Category** | **Condensed code** | **Condensed code frequency** | **Expanded code** | **Expanded code frequency** |
| Positive | Compliment | 112 |  |  |
|  | Organisation | 85 | well organised | 76 |
|  |  |  | timekeeping in sessions | 15 |
|  |  |  | problem solving by organisers | 7 |
|  |  |  | venue | 5 |
|  |  |  | good swag | 4 |
|  |  |  | technical support | 2 |
|  | Personal benefit | 50 | Personal benefit | 48 |
|  |  |  | learnt a lot | 6 |
|  |  |  | will return | 6 |
|  | EDI aspects | 48 | focus on EDI | 38 |
|  |  |  | transport pass | 8 |
|  |  |  | childcare | 5 |
|  |  |  | cost | 5 |
|  |  |  | Trained Awareness Team | 4 |
|  |  |  | signage | 3 |
|  |  |  | grants | 4 |
|  |  |  | quiet room | 3 |
|  | Social aspects | 38 | good atmosphere | 17 |
|  |  |  | good activities (social program) | 16 |
|  |  |  | good participants | 11 |
|  | Academic aspects | 31 | good topics / academic diversity | 11 |
|  |  |  | good talks | 10 |
|  |  |  | plenary talks | 9 |
|  |  |  | good sessions | 11 |
|  | Food | 26 |  |  |
|  | Sustainability | 10 |  |  |
| Negative | Organisation | 59 | tight schedule / inadequate scheduling | 25 |
|  |  |  | inadequate space in room | 20 |
|  |  |  | long days/conference | 19 |
|  |  |  | too many parallel sessions / talks | 16 |
|  |  |  | inadequate communication | 8 |
|  |  |  | inadequate tech | 4 |
|  |  |  | missed printed program | 3 |
| Negative | Organisation | 59 | Problematic sponsor | 1 |
|  | EDI aspects | 57 | inadequate provisions for accessibility | 33 |
|  |  |  | lack of diversity | 10 |
|  |  |  | high costs | 9 |
|  |  |  | inadequate integration / networking of new/alone | 4 |
|  |  |  | issues with travel / venue | 3 |
|  |  |  | inaccessible conference materials | 2 |
|  |  |  | personal pronouns not visible on badges | 2 |
|  |  |  | Visa issues | 2 |
|  |  |  | quiet room | 2 |
|  |  |  | inadequate level of childcare | 1 |
|  | Food | 41 |  |  |
|  | Undesirable interactions | 12 | disrespectful / sexist interactions | 8 |
|  |  |  | unproductive mean questions | 4 |
|  |  |  | intolerance to other ideas | 2 |
|  | COVID | 11 | covid cases | 9 |
|  |  |  | inadequate covid preventative measures | 9 |
|  | Session management (chairs) | 7 |  |  |
|  | Academic aspects | 5 | inadequate academic rigour in talks | 7 |
|  |  |  | homophobic ideas in talks | 1 |
|  |  |  | ideological motivations | 2 |
|  | Sustainability | 4 |  |  |
| Suggestions | Organisation | 38 | alternative scheduling | 21 |
|  |  |  | plan rooms according to expected audience | 6 |
|  |  |  | better communication | 5 |
|  |  |  | hybrid conference | 4 |
|  |  |  | search function in abstracts | 3 |
|  |  |  | better tech | 2 |
|  | Food | 18 |  |  |
|  | DEI aspects | 14 | focus on DEI | 8 |
| Suggestions | DEI aspects | 14 | font/ options on nametag | 3 |
|  |  |  | support for VISAs | 2 |
|  |  |  | registration for part of the conference | 1 |
|  | COVID | 9 |  |  |
|  | Sustainability | 7 | choice of swag | 5 |
|  |  |  | sustainability | 2 |
|  | Session management (chairs) | 4 |  |  |
|  | Social aspects | 3 | themed networking | 3 |
|  | Academic aspects | 1 |  |  |

**Table S12. Models for the observational behavioural data.** This table includes both the research question each model addressed expressed verbally and in lme4 model syntax.

| **Model name** | **Data subset** | **Research question** | **Model formula in lme4 syntax** |
| --- | --- | --- | --- |
| QA.1 | Unmanipulated oral sessions | Do women ask less questions than men do relative to the proportion of the audience who are women? | gender_questioner_female ~ 1 + (1\|session_id / talk_id), offset = logit(audience_women_prop) |
| QA.1c | Conservative unmanipulated oral sessions |  |  |
| QA.1p | Plenary sessions |  | gender_questioner_female ~ 1 + (1\|plenary_id), offset= logit(registration_women_prop), |
| QA.1a-QA.1e | Unmanipulated oral sessions | What conditions can encourage women to ask questions?  a) Gender of the speaker  b) Gender proportion of the audience  c) Gender of the session host  d) Total size of audience  e) Size of room | gender_questioner_female ~ condition + (1\|session_id/talk_id), offset=logit(audience_women_prop) |
| QA.2 |  | Do women raise their hands less often relative to the proportion of the audience who are women? | cbind(hands_women, hands_men) ~ 1 + (1\|session_id/talk_id), offset = logit(audience_women_prop) |
| QA.3 | Unmanipulated oral sessions where at least one woman and one man raised their hand | Do women get chosen less often than men relative to the proportion of people who raised their hand who are women? | gender_questioner_female ~ 1 + (1\|talk_id), offset = logit(hands_prop_women) |

**Table S13. Models for the effect of the gender of the first questioner.** This table includes both the research question each model addressed expressed verbally and in lme4 model syntax.

| **Model name** | **Data subset** | **Research question** | **Model formula in lme4 syntax** |
| --- | --- | --- | --- |
| QA.4.u | Unmanipulated oral sessions minus question 1 | Do women ask less questions than men do relative to the proportion of the audience who are women? | gender_questioner_female ~ - 1 + gender_first_questioner + (1\|session_id / talk_id), offset = logit(audience_women_prop) |
| QA.4.u.2 | Unmanipulated oral sessions only question 2 |  |  |
| QA.5.u | Unmanipulated oral sessions minus question 1 | Do women raise their hands less often relative to the proportion of the audience who are women? | cbind(hands_women, hands_men) ~ - 1 + gender_first_questioner + (1\|session_id/talk_id), offset = logit(audience_women_prop) |
| QA.5.u.2 | Unmanipulated oral sessions only question 2 |  |  |
| QA.6.u | Unmanipulated oral sessions where at least one woman and one man raised their hand minus question 1 | Do women get chosen less often than men relative to the proportion of people who raised their hand who are women? | gender_questioner_female ~ - 1 + gender_first_questioner + (1\|talk_id), offset = logit(hands_prop_women) |
| QA.6.u.2 | Unmanipulated oral sessions where at least one woman and one man raised their hand only question 2 |  |  |
| QA.4.m | Manipulated oral sessions minus question 1 | Do women ask less questions than men do relative to the proportion of the audience who are women? | gender_questioner_female ~ - 1 + condition + (1\|session_id / talk_id), offset = logit(audience_women_prop) |
| QA.4.m.2 | Manipulated oral sessions only question 2 |  |  |
| QA.5.m | Manipulated oral sessions minus question 1 | Do women raise their hands less often relative to the proportion of the audience who are women? | cbind(hands_women, hands_men) ~ - 1 + condition + (1\|session_id/talk_id), offset = logit(audience_women_prop) |
| QA.5.m.2 | Manipulated oral sessions only question 2 |  |  |
| QA.6.m | Manipulated oral sessions where at least one woman and one man raised their hand minus question 1 | Do women get chosen less often than men relative to the proportion of people who raised their hand who are women? | gender_questioner_female ~ - 1 + condition + (1\|talk_id), offset = logit(hands_prop_women) |
| QA.6.m.2 | Manipulated oral sessions where at least one woman and one man raised their hand only question 2 |  |  |

**Table S14. Dependent variables and predictors used to identify other gender disparities in oral sessions.** The results of this analysis are only presented in the Supporting Materials.

| **Dependent variable** | **Predictors** |
| --- | --- |
| Jumping a question | Question number, questioner gender, host gender |
| Speaking longer than your allocated time | Speaker gender |
| Giving a compliment | Question number, questioner gender |
| Receiving a compliment | Speaker gender |
| Asking a critical question | Questioner gender |
| Receiving a critical question | Speaker gender |

**Table S15. Models for the post-congress survey data.** This table includes both the research question each model addressed expressed verbally and in lme4 model syntax.

| **Model** | **Question** | **Formula** |
| --- | --- | --- |
| ***i) Gender effects on question asking motivation and hesitation*** | | |
| **Motivations (“mot_or_hes”)**: Relevance own research, Making voice heard, Interest in topic, Deeper understanding, Appreciate work  **Hesitations (“mot_or_hes”)**: Too introverted, Rather in private, Phrasing, Not clever, No time, No confidence, Misunderstanding, Irrelevance/unimportant, Intimidation setting, Intimidation audience | | |
| PCS.1 | What motivations and hesitations are affected by gender? | mot_or_hes hesitation ~ gender + career |
| PCS.2 | Which motivations and hesitations are predictors of whether a person asked a question at the congress or not? | ask_question ~ mot_or_hes + gender + career |
| ***ii) How do different social identities experience the conference?*** | | |
| **Social identities/controlling variables (“identity”):** LGBTQ+, Nationality, Affiliation, Expat, English comfort, Expertise | | |
| PCS.3 | Which social identities/controlling variables were associated with the statement “felt heard during the conference”? | felt_heard ~ identity |
| PCS.4 | Which social identities/controlling variables were associated with the statement “felt comfortable being myself during the conference”? | be_yourself ~ identity |
| PCS.5 | Which social identities/controlling variables were associated with the statement “felt like I belong in my research field by attending the conference”? | social_belonging ~ identity |
| ***iii) Perception of equity, diversity and inclusivity among congress attendees*** | | |
| **Social identities/controlling variables (“identity”):** Gender, LGBTQ+, Nationality, Affiliation, Expat, English comfort, Age | | |
| PCS.6 | Which social identities/controlling variables were associated with the statement “the conference attendees represented the diversity of researchers in our field”? | diversity ~ identity |
| PCS.7 | Which social identities/controlling variables were associated with the statement “our research field experiences equity, diversity and inclusion related issues”? | edi_issue ~ identity |
| PCS.8 | Which social identities/controlling variables were associated with the statement “the questions asked after the talks were equally divided across genders”? | no_disparity_qa ~ identity |
|  |  |  |
